# Polymorphic Structures and Transitions of Triglycerides and Complex Fats: A Guide to X‐Ray Scattering Approaches

**DOI:** 10.1111/1541-4337.70271

**Published:** 2025-10-05

**Authors:** Julia Seilert, Megan Holdstock, Yoga Pratama, Amin Sadeghpour, Eckhard Flöter, Michael Rappolt

**Affiliations:** ^1^ Department of Food Process Engineering Technische Universität Berlin Berlin Germany; ^2^ School of Food Science and Nutrition University of Leeds Leeds UK; ^3^ Department of Food Technology, Faculty of Animal and Agricultural Sciences Universitas Diponegoro Semarang Indonesia

**Keywords:** phase transition, polymorphs, solid fat content, triglycerides, X‐ray scattering

## Abstract

This contribution provides a comprehensive review of nanostructural aspects in pure triglyceride compared with more complex, real fat systems. Alongside this journey on understanding the crystallization of triglycerides, we provide a practical guide on using X‐ray scattering to its fullest extent. Data from public‐domain literature of monoacid and mixed‐acid saturated and mono‐unsaturated triglycerides are reviewed in great depth. On the nanoscale, lamellar stacking and hydrocarbon tilt angles are discussed, and on the molecular scale, trends in the chain packing density and concomitant geometry changes are explained. Useful tools to evaluate lab‐scale and synchrotron X‐ray scattering data are presented, including (i) electron density profile calculations, decomposing the lamellar repeat distance into the bilayer and monolayer contributions, (ii) refined estimations of the chain tilt angle in the bilayer region, (iii) crystallite size and strain investigations, and (iv) the determination of the area per hydrocarbon chain. Further, the review summarizes representative crystallographic data over the last decades, including extensive lists of long‐ and short‐spacing data. Generalizing the introduced model‐free methods to real fat systems makes it possible to also analyze complex phase transitions and their solid fat content as derived from the wide‐angle scattering. The main aim of this contribution is to provide a robust set of X‐ray scattering methods for a detailed evaluation of polymorphic states and phase transitions supporting research efforts at the interface of academia and industry.

**Abbreviations**
Bu: butyric acid (C4:0)
C: caproic acid (C10:0)
L: lauric acid (C12:0)
M: myristic acid (C14:0)
P: palmitic acid (C16:0)
S: stearic acid (C18:0)
O: oleic acid (C18:1‐ cis (n9))
E: elaidic acid (C18:1‐ trans (n9))
A: arachidic acid (C20:0)
AMF: anhydrous milk fat
EDP: electron density profile
DoC: degree of crystallinity
FWHM: full‐width half maximum
FHRO: fully hydrogenated rapeseed oil
TAG: triacylglycerol or triglyceride
SatOSat: mono‐unsaturated triglyceride
SatOSat+2: mono‐unsaturated triglyceride with the chain on *sn*‐3 position being two carbons longer
Sat‐2OSat: mono‐unsaturated triglyceride with the chain on *sn*‐1 position being two carbons shorter
SFC: solid fat content
SAXS: small‐angle X‐ray scattering
WAXS: wide‐angle X‐ray scattering
SAXD: small‐angle X‐ray diffraction
WAXD: small‐angle X‐ray diffraction
XRS: X‐ray powder scattering
XRD: X‐ray powder diffraction
USAXS: ultra‐small angle X‐ray scattering

## 1 Introduction

The melting and crystallization properties of fats are directed by the constituting triglycerides (triacylglycerols, short TAGs). Over the last century, the nanostructural information within TAG crystals has been investigated and linked to macroscopic properties of fats. In general, small differences in molecular structure, for example, the number of carbon atoms and the degree of saturation, result in significant differences in melting point temperatures. But even for single TAGs, multiple melting point temperatures were communicated by Duffy as early as 1853 (Duffy 1853) and later by Othmer (Othmer 1915). In 1934, the group of Thomas Malkin (Clarkson and Malkin 1934) performed early X‐ray diffraction (XRD) experiments and linked the different melting points to the TAG's polymorphism (note, diffraction is a special case scattering, which refers to the constructive interference of light, that is, revealing the crystalline material; scattering encompasses both diffraction and diffuse scattering). In the years after, many studies followed, expanding the research to other monoacid and mixed acid TAGs—as exemplified by Edwin Lutton, another pioneer on the polymorphism of fat (Lutton 1945, 1948, 1950, 1955a). XRD became an invaluable tool to study crystal structure of different homologous series of TAGs including monoacid TAGs (Hagemann and Tallent 1972; van Langevelde et al. 2001a, 1999), symmetrical (van de Streek et al. 1999; van Langevelde et al. 2000) and asymmetrical (Jong and Soest 1978; van Soest et al. 1990), mixed‐acid TAGs, and homologous series including unsaturated fatty acids (Jong et al. 1991; van Mechelen et al. 2006a, 2006b, 2008a, 2008b). In X‐ray scattering, the wide‐angle region gives information about the polymorphic state (7 < *q* < 20 nm^−1^). From the small‐angle region (0.05 < *q* < 7 nm^−1^), the lamellar stacking (Lutton 1948) and crystallite size (Scherrer 1918) can be determined. More than half a century later, ultra‐small angle scattering (USAXS) emerged, allowing for the determination of TAG crystallite aggregation on larger length scales (Peyronel et al. 2014; Penagos et al. 2024a, 2024b). However, we shall focus in this review on the packing and stacking of TAGs; that is, covering lengths from 0.02 to 20 nm.

The impressive data collection over the last century holds nanostructural similarities for alkyl‐based materials. This is certainly a driver of understanding on a molecular level, given the variation in chain length differences and the degree of saturation. Nonetheless, the translation of pure component data into real fat systems is not a straightforward task, considering the complexity in mixing behavior of binary TAG mixtures already documented as early as 1972 by Knoester et al. (1972) and expanded later in 1990 by Wesdorp (1990), followed by many others.

Real fat systems relevant to the food industry were studied extensively over the last decades. The unique crystallization behavior of palm oil (PO), which was broadly studied (Chen et al. 2002; Smith 2001; Sainlaud et al. 2022; Zaliha et al. 2018; Zhang et al. 2013), triggered research on mono‐unsaturated (Bayés‐García et al. 2013b; Gibon et al. 1986; Macridachis et al. 2025; Minato et al. 1996; Mykhaylyk and Hamley 2004; Sato et al. 1989; Taguchi et al. 2021; van Mechelen et al. 2008a), and polyunsaturated TAGs (Bayés‐García et al. 2011; Lu et al. 2019; Koyano et al. 1992; Watanabe et al. 2018). These are known to form molecular compounds (Bayés‐García et al. 2015, 2024; Nakanishi et al. 2018; Takeuchi et al. 2002a), specifically OPO/POP (Nakanishi et al. 2018). Research on cocoa butter (CB) and cocoa butter equivalents (CBE), and substitutes (CBS) highlighted the role of saturated fatty acid distribution in mono‐unsaturated triglycerides on the crystallization kinetics and miscibility (Da Silva et al. 2019; Dewettinck et al. 2004; Ghazani and Marangoni 2019; MacMillan et al. 2002; Sasaki et al. 2012; Wille and Lutton 1966). Research on anhydrous milk fat (AMF) highlighted the complexity of TAG crystallization in real fat systems (Breitschuh and Flöter, 2002; Cisneros et al. 2006; Grotenhuis et al. 1999; Mazzanti et al. 2009; Pratama et al. 2023; van Aken et al. 1999; Pratama et al. 2021). Wide‐angle XRD has also been a valuable tool in understanding the crystal structure in commercial fats used for spreads and margarines (deMan 1992).

The composition of a fat and the applied process parameters direct the crystallization. Polymorphic transition is straightforward in pure systems or systems of miscible TAGs forming a single solid phase (Sato et al. 2013). Recently, different factors either facilitating or slowing down the phase transition in TAGs were reviewed by Cholakova and Denkov (2024) and Yang et al. (2024). Different crystallization pathways (including sequential crystallization, i.e., fractionation) have been documented in systems of strictly immiscible TAGs. Real fat systems—often characterized by limited miscibility—show a superposition of polymorphic transition and fractionation, and selective co‐crystallization (Seilert et al. 2024a). The molecular makeup of TAGs, that is, chain length mismatches, saturation, and their preferred adaptation to lamellar stacking, plays here a role (Himawan et al. 2006; Wesdorp et al. 2013; Yang et al. 2024). The complexity of the described phenomena certainly hampers the interpretation of X‐ray scattering data of real fat systems. Furthermore, impurities and concomitant lattice disorders further complicate the interpretation of X‐ray data. We note that impurities cause an increase in the area per hydrocarbon chain, a decrease in the tilt‐angle of the chains, and consequently an increase in the long‐spacings of the crystal polymorphs (see Sections 4.2, 5.2, and 3.3). The influence of lattice disorders is discussed together with the evaluation of crystal domain sizes (see Section 4.3). Quantitatively, impurities are best identified by mass spectroscopy (Murphy 2018), and types of lattice disorders can be identified by applying diffraction peak shape analysis (Pabst 2006).

In this study, we review two main research questions. At first, the similarities in the nanostructural stacking and packing information in (pure) triglyceride crystals are reviewed. Therefore, literature data on the TAG homologous series of monoacid saturated, mixed‐acid saturated, and mono‐unsaturated TAGs have been analyzed, and trends in their packing geometries are documented. The calculation of electron density profiles (EDPs), the chain tilt angle determination, crystallite size, and area per chain calculation are described. Further, the concept of subcells is reviewed, and WAXD data for polymorph identification of various triglycerides are summarized. Addressing the second research question, we discuss the comparability of pure component data to structural data of real fat systems. Therefore, the EDP, chain tilt angle, and polymorphic identification for selected fats and related pure components are compared. Methods for solid fat content (SFC) determination of real fat systems via WAXS are summarized. Finally, but importantly, we are providing a practical tool kit of statistically robust and easily applicable methods, supporting a successful polymorph characterization, both concerning static and dynamic analysis of TAG‐based systems.

## 2 Data Review and Acquisition

### 2.1 Literature Data

In this contribution, powder diffraction data serve to illustrate the process of determining the electron density profiles (EDPs) and the tilt angle from SAXS and the subcell packing (area per chain) from WAXS. Thus, data on the unit cell and subcell packing of several TAGs was gathered from a public‐domain literature review (Baker et al. 2014; Bayés‐García et al. 2013a, 2013b, 2015, 2016; Bhaggan et al. 2018a, 2018b; Birker et al. 1991; Bouzidi and Narine 2012; Danthine 2024; Elisabettini et al. 1998; Ghazani and Marangoni 2018; Gibon et al. 1984; Kellens et al. 1990a, 1990b; Kodali et al. 1990, 1987; Lavery 1958; Lavigne et al. 1993; Lutton 1948, 1951, 1955b; Macridachis et al. 2022, 2025; Mykhaylyk and Hamley 2004; Oh et al. 2002; Pratama et al. 2022; Sato et al. 1989, 1999, 2001; Taguchi et al. 2021; Takeuchi et al. 2020, 2002a, 2003, 2002b; Ueno et al. 1997; van Langevelde et al. 2001b, 1999; van Mechelen et al. 2006a, 2006b, 2008a, 2008b, 2008c; Watanabe et al. 2018; Yoshikawa et al. 2022; Zhang et al. 2009). The free software VESTA (Momma and Izumi, 2011) was used to simulate powder X‐ray diffraction patterns for the triglycerides listed in Table 1.

**TABLE 1:** List of triglycerides for which powder X‐ray diffraction patterns were generated using VESTA (Momma & Izumi, 2011), referring to the analysis presented in Figures 3, 4, and 6, 7, 8.

Triglyceride group	TAGs	References
Monoacid saturated TAGs	MMM, SSS	van Langevelde et al. (2001b)
	PPP	van Langevelde et al. (1999)
Mixed acid saturated TAGs	PSP, PSS, PPS, MMP, LMM, LLM	van Mechelen et al. (2008b, 2008c)
Mono‐unsaturated TAGs	SOS	Mykhaylyk and Hamley (2004); van Mechelen et al. (2006b)
	POS, SOA, POP, MOM, LOL	van Mechelen et al. (2006a, 2008b, 2008c)

### 2.2 Lab X‐Ray Scattering Data

Complementing literature data and small‐angle X‐ray scattering (SAXS) and wide‐angle X‐ray scattering (WAXS) data on cocoa butter (CB) were acquired on a lab‐based X‐ray scattering instrument. Experimental details on the setup and the standard data reduction applied can be found elsewhere (Seilert et al. 2024a; Pratama et al. 2021). CB was melted on a hot plate prior to injection into quartz disposable capillaries with an outside diameter of 1.5 mm and sealed with wax. After keeping CB at 80°C for 10 min, it was cooled down to 20°C at a rate of 3°C/min. The X‐ray patterns were collected during the subsequent isothermal holding time. During the isothermal hold, exposure times of 60 s were applied, guaranteeing an optimum counting rate. Data analysis was performed using OriginPro 2022b.

## 3 Literature Review on Molecular Structures of Triglyceride Crystals

### 3.1 Molecular Structures and Polymorphism

The major force stabilizing a TAG crystal is short‐range London‐van der Waals forces (Coupland 2014; Marangoni 2012). TAGs are mainly composed of alkyl chains, which are nonpolar but polarizable, and, thus, the short‐range London dispersion force between individual atoms and molecules is quite weak and decreases quickly with separation, that is, with the sixth power of the distance (Coupland 2014). The glycerol backbone possesses polarized bonds so that additionally long‐range induction forces must be accounted for (Pink 2019).

Depending on the alkyl chains, that is, their difference in chain lengths and saturation, triglycerides commonly arrange in either double (2L) or triple (3L) chain length stacking (Himawan et al. 2006). 2L stacking repeat distances are in the order of 45 to 50 Å, and for 3L stacking distances lie in the range of 65 to 75 Å—this is captured in the SAXS region. In 3L stacking, one alkyl chain forms a monolayer, whereas the other two alkyl chains form a bilayer. Jointly, they define the lamellar repeat distance. Depending on the (quasi) long‐range stacking order and the different EDPs given for 2L or 3L stacking, up to seven orders can be recorded (Mykhaylyk and Hamley 2004).

Like for related alkyl‐chain molecules, the crystal packing of triglycerides is directed by the hydrocarbon chains. These allow various molecular arrangements, that is, polymorphism. Different from *n*‐alkanes, triglycerides exhibit monotropic polymorphism with only one stable phase over the entire temperature range. This also means that spontaneous phase transitions only occur from less stable to more stable polymorphs. These transitions often require a long time, and metastable phases are initially formed rather than the thermodynamically stable polymorph (Himawan et al. 2006; Sato et al. 2013), following Ostwald's rule of stages (Ostwald 1897). The behavior differs for different TAGs. Monoacid TAGs undergo a direct polymorphic transition from the α phase into the most stable form β, sometimes passing a short‐lived β′ phase (Bayés‐García et al. 2024; Kellens et al. 1990a; Sato and Kuroda 1987). Some saturated mixed‐acid TAGs show multiple β′ phases (Danthine 2024; Kodali et al. 1990, 1989; Sato et al. 2001), some do not form the β polymorph like PPM (Sato et al. 2001) and symmetrical mixed‐acid TAGs (C_n_.C_n+2_.C_n_) (van Langevelde et al. 2000). Mono‐unsaturated TAGs like POP, SOS, POS exhibit multiple polymorphic forms. This has been extensively documented (Baker et al. 2014; Bayés‐García et al. 2013b; Gibon et al. 1986; Sato et al. 1989; Taguchi et al. 2021; Ueno et al. 1997).

The long spacings change with the polymorphic form, the chain length, and depend also on the chain tilt with respect to the layer normal. For a pure component of known carbon number, *N*_C_, the long spacing can be used to determine the tilt angle by applying basic trigonometry. This is explored in Section 4.2. In a mixed solid phase, the *d*‐spacing reflects the structure of the predominant phase present—either a mixed crystal or co‐existing solid phases of similar molecular makeup. In the latter case, when the TAGs are very similar in their molecular build‐up, an assignment of all reflections to the given polymorphs is difficult.

### 3.2 The Role of the Glycerol Moiety

**FIGURE 1:**
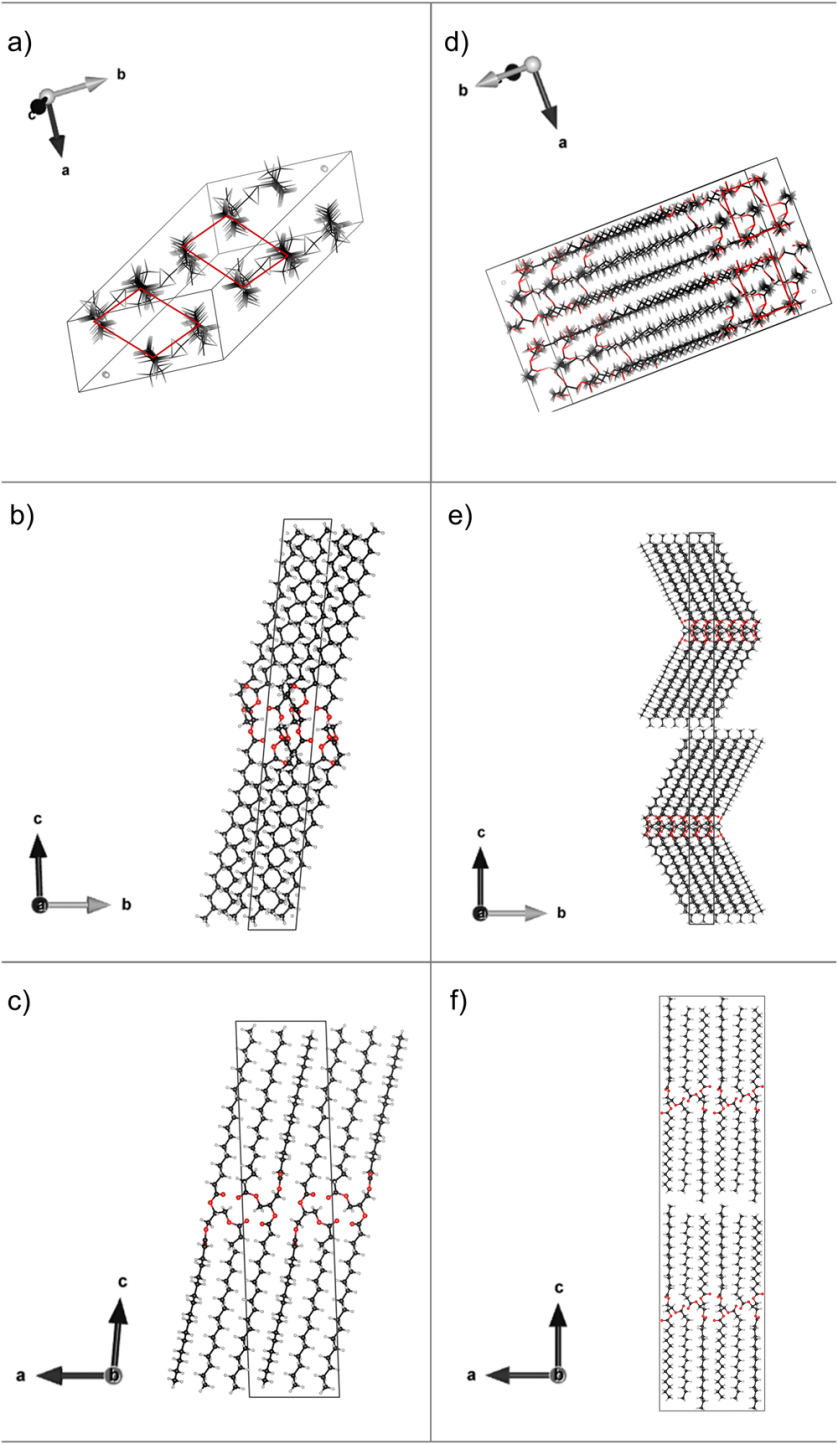
PSS in β‐polymorph displaying the (a) subcell, (b) bc‐plane, and (c) ac‐plane. PSS in the β′‐polymorph illustrating the (d) subcell, (e) bc‐plane, and (f) ac‐plane. Carbons are color‐coded in black, oxygen in red, hydrogen in grey, subcells indicated by red lines, and the unit cells are indicated by a black box. XRD data from van Mechelen et al. (2008b, 2008c) are visualized by using VESTA (Momma and Izumi, 2011).

In TAG crystals, the glycerol backbone adapts two conformations, namely the tuning fork and the chair. In the tuning fork configuration, the fatty acids on the *sn‐*1 position and the *sn*‐3 position are adjacent to each other, meaning the hydrocarbon chains are packed in the same direction with respect to the glycerol backbone, while the fatty acid on the *sn*‐2 position packs in the opposite direction. In the chair configuration, either the fatty acids that are on the *sn*‐1‐position and *sn*‐3‐position or those on the *sn*‐2 position and *sn*‐3 position are adjacent (Sato 2018b). Within a TAG crystal, the glycerol moiety builds a layer that is parallel to the lamellar interface. Table 2 summarizes the glycerol configuration of some triglycerides. Regarding pure unsaturated TAGs, there is a general understanding that unsaturated fatty acids do not form a leaflet with the saturated fatty acids in more stable polymorphic forms. From the minimization of the potential energy of the functional groups of the TAGs, e.g., the hydrocarbon chains, it can be deduced that the unsaturated fatty acids are organized in one leaflet which is separated from the leaflets formed by saturated fatty acids (Kodali et al. 1987; Sato 2001; van Mechelen et al. 2006a, 2006b, 2008b, 2008c). A common assumption regarding the glycerol configuration of saturated TAGs is that the position of the fatty acids on the glycerol and, thus, the chirality of the TAG is the determining factor (Sato 2018a). Accordingly, for pure saturated TAGs, an achiral or symmetric TAG adapts the tuning fork configuration, while a pure asymmetric TAG always adapts the chair configuration. However, there are exceptions to this rule, considering the triglyceride PSS, an asymmetric triglyceride, which adapts the chair configuration in the β′‐polymorph (Figure 1d–f). Interestingly, PSS adapts the turning fork configuration in the thermodynamically most stable *β*‐polymorph (Figure 1a–c) (van Mechelen et al. 2008b). PSP, the symmetric counterparts, show a similar behavior.

**TABLE 2:** Summary of reported glycerol configurations of triglycerides in β′ and β‐polymorph.

TAG	Polymorph	Configuration	References	TAG	Polymorph	Configuration	References
Saturated							
Asymmetric				Symmetric			
PSS	β	Tuning fork	van Mechelen et al. (2008a)	PSP	β	Tuning fork	van Mechelen et al. (2008b)
PPS	β	Tuning fork	van Mechelenet al. (2008a)	CCC	β	Tuning fork	Birker et al. (1991); van Langevelde et al. (1999)
MMP	β	Tuning fork	van Mechelen et al. (2008a)	LLL	β	Tuning fork	van Langevelde et al. (1999)
LLM	β	Tuning fork	van Mechelen et al. (2008a)	MMM	β	Tuning fork	van Langevelde et al. (2001b)
MML	β	Tuning fork	van Mechelen et al. (2008a)	PPP	β	Tuning fork	van Langevelde et al. (1999)
MPP	β′	Chair	Sato et al. (2001)	LML	β′	Chair	Birker et al. (1991)
PSS	β′	Chair	van Mechelenet al. (2008b)	PSP	β′	Chair	Birker et al. (1991); van Mechelen et al. (2008c)
PPS	β′	Chair	van Mechelen et al. (2008b)	CLC	β′	Chair	van Langevelde et al. (1999)
BuSP	β′	Chair	Pratama et al. (2022)	MPM	β′	Chair	van Langevelde et al. (1999)
Unsaturated							
Asymmetric				Symmetric			
PPE	β	Tuning fork	van Mechelenet al. (2008a)	PEP	β	Tuning fork	van Mechelen et al. (2008b)
SSE	β	Tuning fork	van Mechelenet al. (2008a)	SES	β	Tuning fork	van Mechelen et al. (2008b)
PPE	β′	Chair	van Mechelen et al. (2008b)	LOL	β	Tuning fork	van Mechelen et al. (2008a)
POS	β	Tuning fork	van Mechelen et al. (2006a)	MOM	β	Tuning fork	van Mechelen et al. (2006b)
SOA	β	Tuning fork	van Mechelen et al. (2006a)	POP	β	Tuning fork	van Mechelen et al. (2006b)
PPO	β	Chair	Taguchi et al. (2021)	SOS	β	Tuning fork	van Mechelen et al. (2006b)
SSO	β	Chair	Watanabe et al. (2018)	LOL	β′	Chair	van Mechelen et al. (2008c)
				OEO	β	Tuning fork	Kodali et al. (1987)
				OEO	β′	Tuning fork	Kodali et al. (1987)
				OSO	β	Tuning fork	Kodali et al. (1987)
				OSO	β′	Tuning fork	Kodali et al. (1987)

The terminal methyl groups of the alkyl chains form the methyl end plane. It accounts for the chain length differences, mismatches of hydrocarbon chains packed in the same direction, and the chain inclination of the respective polymorph. Differences in the chain lengths of adjacent fatty acids cause a distorted lamellar interface. Resulting voids or cavities decrease the local crystal density (Wesdorp et al. 2013). According to Hernqvist, an irregular methyl end group region is formed in the α‐polymorph (Hernqvist 1990). This allows a higher degree of mobility (“liquid‐like”), which induces a rapid transition from the *α*‐modification into a more stable polymorph, and can be compared with a rotator phase in *n*‐alkanes. This could be confirmed via modeling the electron density profile of cocoa in the α‐polymorph (Ladd Parada et al. 2018). Interestingly, multiple α‐phases were observed for PPO starting from α‐2L with a long spacing of 48 Å, followed by another α‐2L phase with a long spacing of 46 Å, and an α‐3L phase with a long spacing of 78 Å. The tuning fork configuration is presumed in the short‐lived α‐2L phase (46 Å) (Taguchi et al. 2021).

In the β′‐polymorph, the methyl end plane is more regularly packed as compared with the α‐polymorph due to an increased density in chain packing accomplished by chain tilt of about 30°. In the β‐form, the hydrocarbon chains are even more densely packed, resulting in a relatively even methyl end plane and an increased tilt angle of about 33° (Hernqvist 1990). De Jong and van Soest (1978) and Wesdorp et al. (2013) describe the methyl end plane as a terrace with steps differing in their positions on the terrace for different subcategories of TAGs. Hence, the terminal methyl end groups do not lie on a straight line and do not necessarily form an even plane but rather a boundary region with a particular structure.

### 3.3 Triglyceride Unit Cell Information

The periodic arrangement of atoms in space is described by the unit cell. Unit cells are the fundamental building blocks of a crystal. They contain the smallest group of atoms or molecules constituting the crystal by repetition (Idziak 2018). X‐ray diffraction techniques have been widely employed to study crystal structures and polymorphic behavior. Various research groups have fully determined TAG‐based crystal structures, including the unit cell determination, applying powder X‐ray diffraction (XRD) and numerical refinement of the data. Powder X‐ray measurements are the most common in studying lipid crystals, with only a few instances of single‐crystal studies (Vand 1951; Sato et al. 2001; Skoda et al. 1967; Jensen and Mabis 1963, 1966; van Langevelde et al. 2000). Single‐crystal X‐ray diffraction provides highly accurate structural data but requires well‐formed single crystals, which are difficult to grow. In contrast, powder X‐ray diffraction, a sample containing many randomly oriented crystals is analyzed, offering qualitative insights into the dominant crystal structure.

For the α polymorph, a hexagonal unit cell is generally reported. Note, due to the free rotation of the hydrocarbon chains in this phase, the apparent molecular shape is a cylinder, in which the hexagonal molecular packing is the closest possible arrangement. For the metastable polymorph β′, a monoclinic unit cell is reported. For the most stable polymorphic form, β, triclinic and monoclinic unit cells are reported. Representative unit cell data are summarized in Table 3.

**TABLE 3:** Summary of unit cell parameters of selected triglycerides.

TAG	Poly‐morph	Unit cell type, space group, and no. of molecules	Unit cell parameters		References
			a, b, c (Å)	α, β, γ (°)	
Monoacid					
SSS	β‐2L	Triclinic, $P\bar{1}$, *Z* = 2	12.0053, 51.902, 5.4450	73.752, 100.256, 117.691	van Langevelde et al. (2001b)
PPP	β‐2L	Triclinic, $P\bar{1}$, *Z* = 2	5.4514, 11.945, 40.482	84.662, 86.97, 79.77	van Langevelde et al. (1999)
MMM	β‐2L	Triclinic, $P\bar{1}$, *Z* = 2	12.0626, 47.714, 5.4588	73.388, 100.408, 118.274	van Langevelde et al. (2001b)
LLL	β‐2L	Triclinic, $P\bar{1}$, *Z* = 2	12.084, 31.617, 5.468	94.82, 100.45, 96.41	Gibon et al. (1984)
Mixed‐acid					
PPM	β′‐2L	Monoclinic, C2, *Z* = 8	16.534, 7.537, 81.626	90, 90.28, 90	Sato et al. (2001)
PSP	β′‐2L	Monoclinic, I2, *Z* = 4	22.253, 5.634, 85.263	90, 90.80, 90	van Mechelen et al. (2008c)
PSS	β_0_’‐2L	Monoclinic, I2, *Z* = 4	22.651, 5.653, 89.462	90, 90.01, 90	van Mechelen et al. (2008c)
PPS	β′‐2L	Monoclinic, I2, *Z* = 4	22.751, 5.650, 86.746	90, 93.968, 90	van Mechelen et al. (2008c)
PSP	β‐2L	Triclinic, $P\bar{1}$, *Z* = 2	5.439, 12.18, 41.60	88.73, 93.10, 99.97	van Mechelen et al. (2008b)
PSS	β‐2L	Triclinic, $P\bar{1}$, *Z* = 2	5.412, 11.14, 46.45	91.49, 94.85, 96.75	van Mechelen et al. (2008b)
PPS	β‐2L	Triclinic, $P\bar{1}$, *Z* = 2	5.437, 11.92, 41.93	88.18, 91.15, 100.02	van Mechelen et al. (2008b)
MMP	β‐2L	Triclinic, $P\bar{1}$, *Z* = 2	5.457, 12.14, 37.37	92.32, 88.79, 100.45	van Mechelen et al. (2008b)
LMM	β‐2L	Triclinic, $P\bar{1}$, *Z* = 2	5.444, 11.45, 36.70	90.79, 95.52, 97.18	van Mechelen et al. (2008b)
LLM	β‐2L	Triclinic, $P\bar{1}$, *Z* = 2	5.460, 12.15, 33.05	96.19, 87.05, 100.48	van Mechelen et al. (2008b)
Mono‐unsaturated					
LOL	β′‐2L	Triclinic, $P\bar{1}$, *Z* = 2	12.05, 36.59, 5.427	95, 101.5, 84.6	van Mechelen et al. (2008c)
LOL	β_1_‐3L^a^	Monoclinic, Cc, *Z* = 4	5.449, 104.42, 8.143	90, 88.5, 90	van Mechelen et al. (2008c)
MOM	β_1_‐3L	Monoclinic, $P2_{1}/n$, *Z* = 4	5.453, 112.75, 8.195	90, 88.84, 90	van Mechelen et al. (2006a)
POP	β_1_‐3L	Monoclinic, $P2_{1}/n$, *Z* = 4	5.450, 121.32, 8.209	90, 88.85, 90	van Mechelen et al. (2006a)
POS	β_1_‐3L	Monoclinic, $P2_{1}/n$, *Z* = 4	5.445, 125.98, 8.195	90, 88.79, 90	van Mechelen et al. (2006a)
SOS	β_1_‐3L	Monoclinic, $P2_{1}/n$, *Z* = 4	5.442, 129.90, 8.184	90, 88.71, 90	van Mechelen et al. (2006a)
LOL	β_2_‐3L	Triclinic, $P\bar{1}$, *Z* = 2	5.45, 7.736, 46.10	81.5, 89.7, 90.2	van Mechelen et al. (2008a)
POP	β_2_‐3L	Monoclinic, Cc, *Z* = 4	5.447, 122.62, 8.220	90, 88.78, 90	van Mechelen et al. (2006b)
POS	β_2_‐3L	Monoclinic, Cc, *Z* = 4	5.424, 126.53, 8.121	90, 88.51, 90	van Mechelen et al. (2006b)
SOS	β_2_‐3L	Monoclinic, Cc, *Z* = 4	5.440, 130.30, 8.221	90, 88.75, 90	van Mechelen et al. (2006b)

*Note*: Z = number of molecules in a unit cell.

^a^ The subscripts relate to the stability of the polymorphic form, for example, the melting temperature of *β*_1_ is greater than that of *β*_2_.

In a homologous series of triglycerides, for example, monoacid TAGs, C_n_C_n_C_n_‐type, or symmetrical mono‐unsaturated TAGs (SatOSat), the unit cell types are identical. This includes LLL, MMM, PPP, and SSS in β‐2L assume a triclinic unit cell with *Z* = 2 molecules with the space group $P\bar{1}$. Similarly, LOL, MOM, POP, and SOS in β_1_‐3L (also known as form VI) assume a monoclinic subcell with *Z* = 4 molecules with the space group $P2_{1}/n$. In the β_2_‐3L polymorph (form V), POP, POS, and SOS assume a monoclinic unit cell, with the exception of LOL forming a triclinic subcell. Note, the full geometrical description of the space groups can be found in the International Tables for Crystallography (2016).

## 4 Nanostructural Stacking Information Deduced From SAXD

Different types of nanostructural information are derived from SAXD. Each diffraction peak contains four different types of information (Li et al. 2017). These concern (i) the lattice repeat distance, (ii) the electron density contrast referring to a given repeat distance, (iii) the crystal nanoplatelet size and strain, and (iv) the type of lattice disorder, which can be derived from the peak position, the peak height or intensity, the peak width, and the peak shape, respectively. The most common in XRD analyses of TAGs is based on the diffraction peak position from which the *d*‐spacings or lattice spacing (also referred to as long spacings in SAXD or short spacings in WAXD). Even though we are not further discussing peak shape analysis in this review, it is worth mentioning that polymorphs of TAG mixtures and/or polymorphs with an higher amount of impurities compromise the crystal quality often displaying only quasi‐long range order, which is displayed in the broadening of the diffraction peaks by increasing order (see Figure S2), which is also known as second type of lattice disorder (for a review see Pabst 2006). These diffraction peaks are best fitted with Lorentzian peak‐shaped distributions, while the β′ and β‐phases of pure TAGs may, in contrast, display thermal disorder (note here the peak widths remain constant), in which the diffraction peaks are most appropriately fitted with Gaussian distributions. Those who are not interested in these finer lattice disorder details should use Pearson VII distributions for practical reasons. This function covers all peak shapes from Lorentzian to Gaussian. Details on the information held in the peak intensity (i.e., electron density contrast) and the peak width (crystallite size and strain) are discussed in the following two sections.

### 4.1 Calculation of Electron Density Profiles from TAG Polymorphs by the Classical Fourier Analysis

The projection of the electron density along the layer normal can be calculated for structures by applying a classical Fourier analysis. The resulting electron density profile (EDP) allows identification of the position of glycerol groups and the methyl end plane, tilt angles of the hydrocarbon chains, as well as the thicknesses of the mono‐ and bilayers within 3L‐stacking composition of TAGs. The EDP can be reconstructed from the first few lamellar peaks measured in the SAXS regime. This approach has previously been applied to analyze lipid bilayer structures (Blaurock 1982; Levine et al. 1968; Li et al. 2017; Nagle and Tristram‐Nagle 2000; Rappolt 2010; Torbet and Wilkins, 1976; Tristram‐Nagle et al. 2002; Wilkins et al. 1971), but also adapted to study the polymorphism and phase transitions of (pure) triglycerides (Mykhaylyk et al. 2007; Mykhaylyk and Hamley 2004; Pratama et al. 2022) and fats, for example, cocoa butter in the liquid and solid phase (Ladd Parada et al. 2018; Sadeghpour et al. 2018; Simone et al. 2024), and (buffalo) milk fat (Pratama et al. 2021).

**FIGURE 2:**
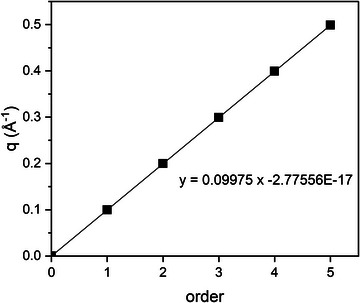
Linear regression performed on peak positions *q* up to the 5th order for POS in β_1_‐3L (data from van Mechelen et al. (2006a) and reconstructed in VESTA), including *h* = 0. From the slope, the *d*‐spacing (average long spacing) of 62.99 Å is calculated.

EDPs can be determined from the position and intensities of the Bragg diffraction peaks in the small‐angle region (0.05 < *q* < 7 nm^−1^). This includes:

1. Fit each reflection to obtain the peak position, *q*_h_, and intensity (peak area), *I*_h_.
2. Correct the reflection intensities by the so‐called Lorentz correction (*L*_C_), yielding *I*_h,corr_ = *L*_C_·*I*_h_. The Lorentz correction of the reflection intensities accounts for the relative differences in the diffraction probabilities of different crystal planes to diffract (Blaurock 1982). In nonoriented, “powder‐like” samples, e.g., triglyceride mixtures, crystal planes with greater *d*‐spacings display relatively higher diffraction probabilities than those with smaller *d*‐spacings. Commonly, the geometry of the instrumental setup is also integrated in this correction. In brief, for an ideal point focus and an ideal line focus, *L*_C_ = *h* ^2^ and *h*, respectively, where *h* is the diffraction order. We note that our used setup (SAXSpace instrument from Anton Paar, Austria) is neither reflecting an ideal point nor ideal line focus, and we empirically determined a correction factor of *h* ^1.5^ in this specific case (Pratama et al. 2021).
3. Taking the square root of the corrected intensities, *I*_h,corr_, gives the amplitudes *F*_h_ of the electron contrast variations. Note, low intensities reflect low contrast contributions, and vice versa, high intensities reflect strong contrast contributions.
4. Calculating the EDP via a Fourier transform, Equation (1). In the case of centrosymmetric EDPs, the Fourier transform is obtained by the summation of cosine terms only:
(1) $$\Delta \rho \left(x\right)=\sum_{h=1}^{h_{max}}\alpha_{h}F_{h}\cos\left(\frac{2\pi xh}{d}\right),$$

where Δρ is the electron density contrast, *h* the Miller index (diffraction order), $\alpha_{h}$ the phase factors, *x* the real space variable, and *d* the lattice spacing. The lattice spacing, *d*, is determined from the peak positions *q*_h_ = 2π·*h*/*d*, applying a linear regression on the measured peak positions 1/*d*, 2/*d*, …, *h*/*d* and including the direct beam position, 0 (zeroth order) (Figure 2).

Equation (1) only contains a cosine term (no sine term) characteristic of centrosymmetric structures (note, the cosine function is centrosymmetric). This is valid for lipids forming lamellar phases and for triglyceride polymorphs (Li et al. 2017; Mykhaylyk and Hamley 2004). As a consequence, the unknown phases are either +π or −π, and the phase factors *α*_h_ = cos(±π) can only take the values ±1. The phase factors are not the same for every *h*. The phase factors $\alpha_{h}$ for different *h* for 2L‐ and 3L‐phases are summarized in Table 4. Note, that $\alpha_{h}$ is fixed to −1 for *h* = 1, which defines the methyl end plane to be placed at *x* = 0.

**TABLE 4:** Summary of phase signs for Miller indices *h* in 2 and 3L packing according to Mykhaylyk and Hamley (2004).

h	α_h_ (2L)	α_h_ (3L)
1	−1	−1
2	+1^a^	−1
3	−1	+1
4	+/−1^a^	−1
5	−1^b^	−1
6	n.d.	+1
7	n.d.	+1

^a^ Very weak.

^b^ Negative, synchrotron data on CB in the α‐phase (Ladd Parada et al. 2018; Figure S2).

**FIGURE 3:**
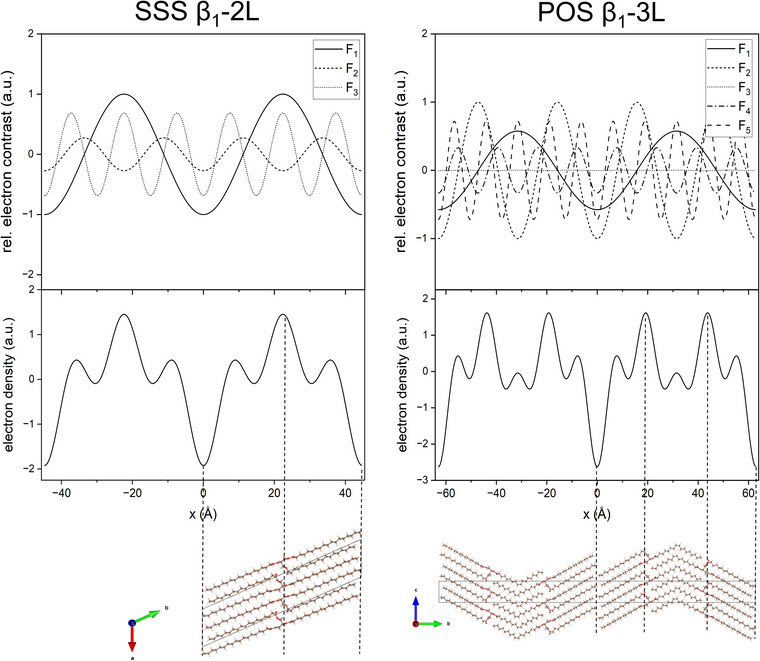
Electron density contrasts (top), resulting electron density profiles (middle), and molecular schemes deduced from crystallography data (bottom) for SSS β_1_‐2L and POS β_1_‐3L.

**FIGURE 4:**
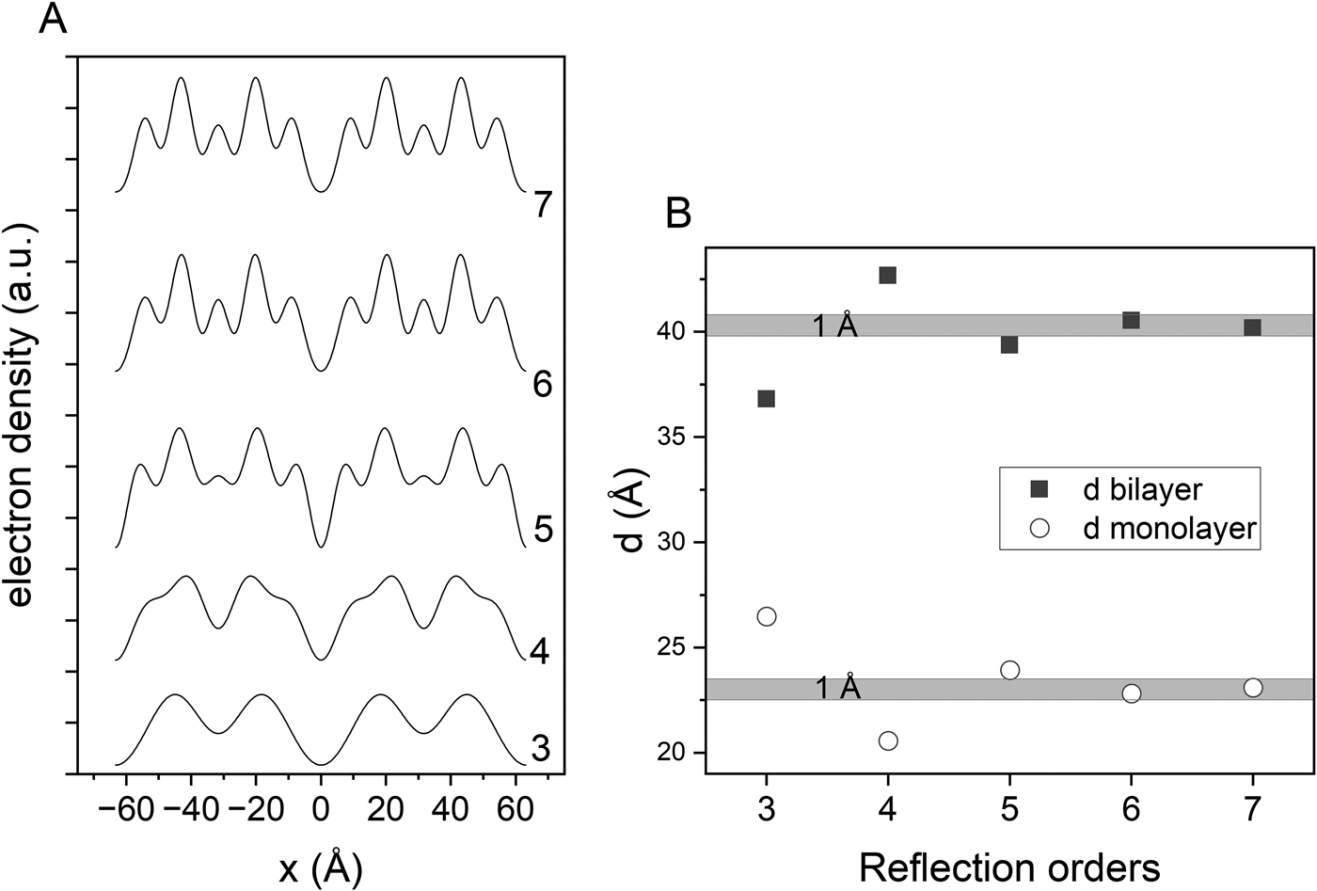
(A) Electron density profiles determined for POS in β_2_‐3L, using an increasing number of reflections as indicated. The *F*_h_ values have been generated using VESTA (Momma and Izumi 2011), and the data are taken from van Mechelen et al. (2006a). (B) Corresponding monolayer and bilayer thickness (*d*_M_ and *d*_B_) as a function of included orders.

The contribution of each electron density contrast, *F*_h_, to the EDP is illustrated in Figure 3 for SSS in β_1_‐2L and POS in β_1_‐3L. The resolution increases with the number of reflections. This is demonstrated in Figure 4, giving the constructed EDPs for POS in β_2_‐3L. In general, reflections of higher order are necessary to obtain a meaningful EDP, that is, determining the bilayer thickness with a precision of ±1Å. It is worth noting that not all consecutive reflections contribute to an EDP. For example, in 2L‐phases, the 2nd order reflection is very weak and, hence, contributes to the EDP only marginally. The same is the case for the 1st and 4th orders in the 3L‐phase, if the triglyceride assumes the γ polymorphic form. Constructing the EDP for SOS in γ‐3L, including all five orders, results in a monolayer and bilayer thickness of 28.7 Å and 43.1 Å, respectively. When omitting the 1st and 4th orders, the mono and bilayer thicknesses are 29.3 Å and 42.5 Å, which are the same within the 1 Å error.

The EDPs differ for different triglyceride categories, monoacid TAGs, unsaturated TAGs, and short‐chain TAGs are demonstrated for SSS in β‐2L, POS in β_1_‐3L, LOL in β′‐2L, β′‐3L, and β‐3L, BuSP in α‐3L and β′‐3L, and SOS γ‐3L (Figure S1). A summary of structural parameters derived from EDPs constructed for monoacid saturated, mixed‐acid saturated, short‐chain saturated, and mono‐unsaturated TAGs is given in Table 5.

**TABLE 5:** Summary of structural information for various triglycerides, the determined long spacings, and bilayer and monolayer thickness.

TAG	Polymorph and lamellar stacking	Long spacing (Å)	*d*_B_ (Å)	*d*_M_ (Å)	Phase signs adopted from Mykhaylyk and Hamley (2004), if not indicated otherwise	References
Mono‐acid saturated TAGs						
MMM	β‐2L	35.74	35.74	n.a.	(−1, +1, −1, −1, −1)	van Langevelde et al. (2001b)
PPP	β‐2L	40.28	40.28	n.a.	(−1, +1, −1, −1, −1)	van Langevelde et al. (1999)
SSS	β‐2L	44.75	44.75	n.a.	(−1, +1, −1, −1, −1)	van Langevelde et al. (2001b)
Mixed‐acid saturated TAGs						
PSP	β′‐2L	42.63	42.63	n.a.	(−1, +1, −1, ‐1, −1)	van Mechelen et al. (2008b)
PSP	β‐2L	41.53	41.53	n.a.	(−1, +1, −1, −1, −1)	van Mechelen et al. (2008a)
PSS	β′‐2L	44.72	44.72	n.a.	(−1, +1, −1, −1, −1)	van Mechelen et al. (2008b)
PSS	β‐2L	46.27	46.27	n.a.	(−1, +1, −1, −1, −1)	van Mechelen et al. (2008a)
PPS	β‐2L	41.92	41.92	n.a.	(−1, +1, −1, −1, −1)	van Mechelen et al. (2008a)
MMP	β‐2L	37.56	37.56	n.a.	(−1, +1, −1, −1, −1)	van Mechelen et al. (2008a)
LMM	β‐2L	36.51	36.51	n.a.	(−1, +1, −1, −1, −1)	van Mechelen et al. (2008a)
LLM	β‐2L	32.83	32.83	n.a.	(−1, +1, −1, −1, −1)	van Mechelen et al. (2008a)
Short‐chain saturated TAGs						
BuSP	α‐3L	56.90	45	11.90	(−1, −1, +1, −1, −1, −1)	Pratama et al. (2022)
BuSP	β′‐3L	51.20	40.6	10.60	(−1, −1, +1, −1, −1, −1)	Pratama et al. (2022)
Mono‐unsaturated TAGs						
POS	β_1_‐3L	62.96	39.29	23.67	(−1, −1, +1, −1, −1)	van Mechelen et al. (2006a)
POS	β_2_‐3L	63.27	39.48	23.79	(−1, −1, +1, −1, −1)	van Mechelen et al. (2006b)
SOS	α_2_‐3L	54.30	29.10	25.20	(−1, −1, +1, +1)^a^	Mykhaylyk and Hamley (2004)
SOS	α_1_‐2L	49.10	49.10	n.a.	(−1, +1, −1)	Mykhaylyk and Hamley (2004)
SOS	γ‐3L	71.80	43.08	28.72	(−1, −1, +1, −1, −1)	Mykhaylyk and Hamley (2004)
SOS	β′‐3L	68.90	42.44	26.46	(−1, −1, +1, −1, −1)	Mykhaylyk and Hamley (2004)
SOS	β_1_‐3L	64.50	40.25	24.25	(−1, −1, +1, −1, −1)	Mykhaylyk and Hamley (2004)
SOS	β_2_‐3L	65.17	40.58	24.60	(−1, −1, +1, −1, −1)	van Mechelen et al. (2006b)
SOA	β_1_‐3L	67.34	41.48	25.86	(−1, −1, +1, −1, −1)	van Mechelen et al. (2006a)
SOA	β_2_‐3L	67.63	42.74	24.89	(−1, −1, +1, −1, −1)	van Mechelen et al. (2006b)
POP	β_1_‐3L	60.65	36.87	23.77	(−1, −1, +1, −1, −1)	van Mechelen et al. (2006a)
POP	β_2_‐3L	60.82	36.98	23.84	(−1, −1, +1, −1, −1)	van Mechelen et al. (2006b)
MOM	β_1_‐3L	56.35	33.36	22.99	(−1, −1, +1, −1, −1)	van Mechelen et al. (2006a)
LOL^b^	β′‐3L	55.46	35.94	19.52	(−1, −1, +1, −1, −1)	van Mechelen et al. (2008a)
LOL	β′_1_‐2L	36.34	36.34	n.a.	(−1, +1, −1, +1, −1)^c^	van Mechelen et al. (2008a)
LOL	β_2_‐3L	52.23	30.92	21.31	(−1, −1, +1, −1, −1)	van Mecheken et al. (2008a)

^a^ Phase signs taken from Mykhaylyk et al. (2007).

^b^ The thermodynamic stability according to van Mechelen et al. (2008a) is β′‐3L < β′_1_‐2L < β_2_‐3L.

^c^ Gave best results in matching EDP construction with unit cell dimensions.

We note that EDPs can also be determined in the same way for mixtures such as milk fat (Pratama et al. 2021) or cocoa butter (Ladd Parada et al. 2018; Sadeghpour et al. 2018; Simone et al. 2024), with the latter containing mainly the three triglycerides, POS, SOS, and POP (see Section 7.2).

### 4.2 Chain Tilt Angle Determination from EDP Data

**FIGURE 5:**
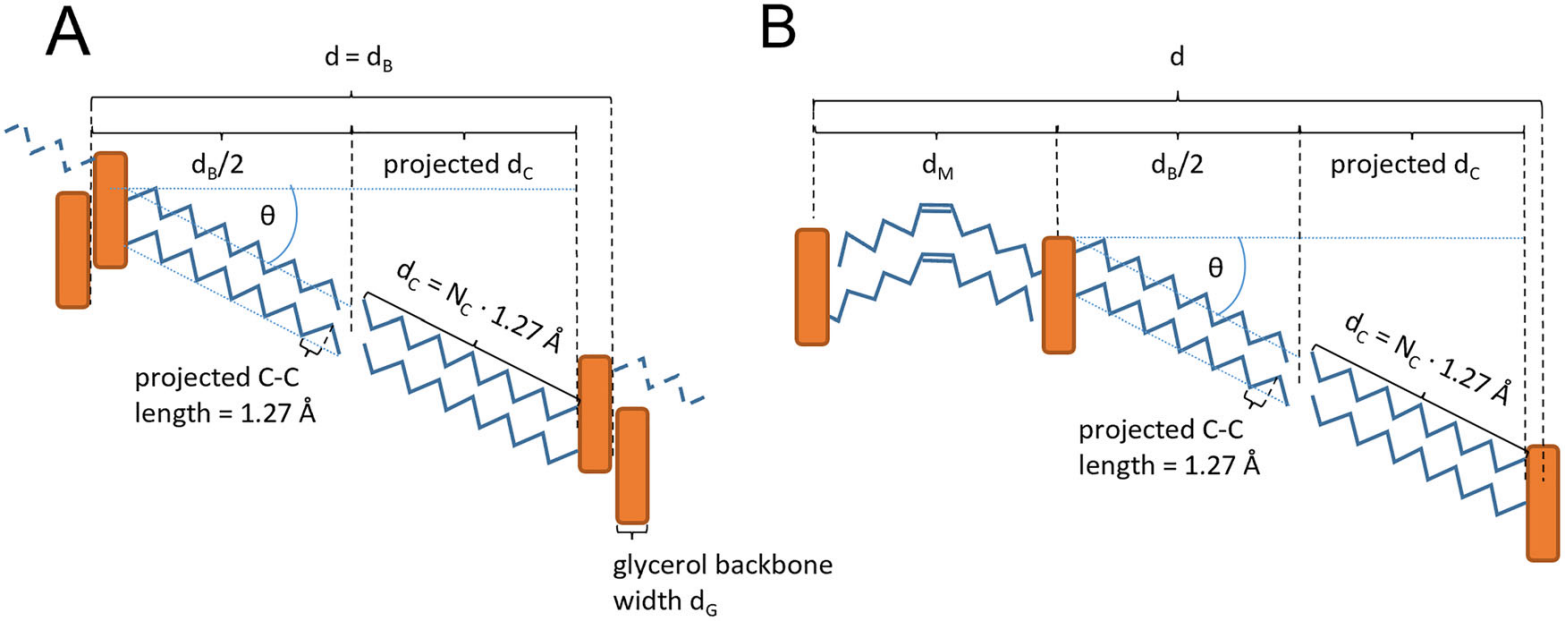
Tilt angle estimation given for 2L‐polymorphs (a) and 3L‐polymorphs (b). Hydrocarbon chains are color‐coded in blue, and the glycerol backbones in orange.

**FIGURE 6:**
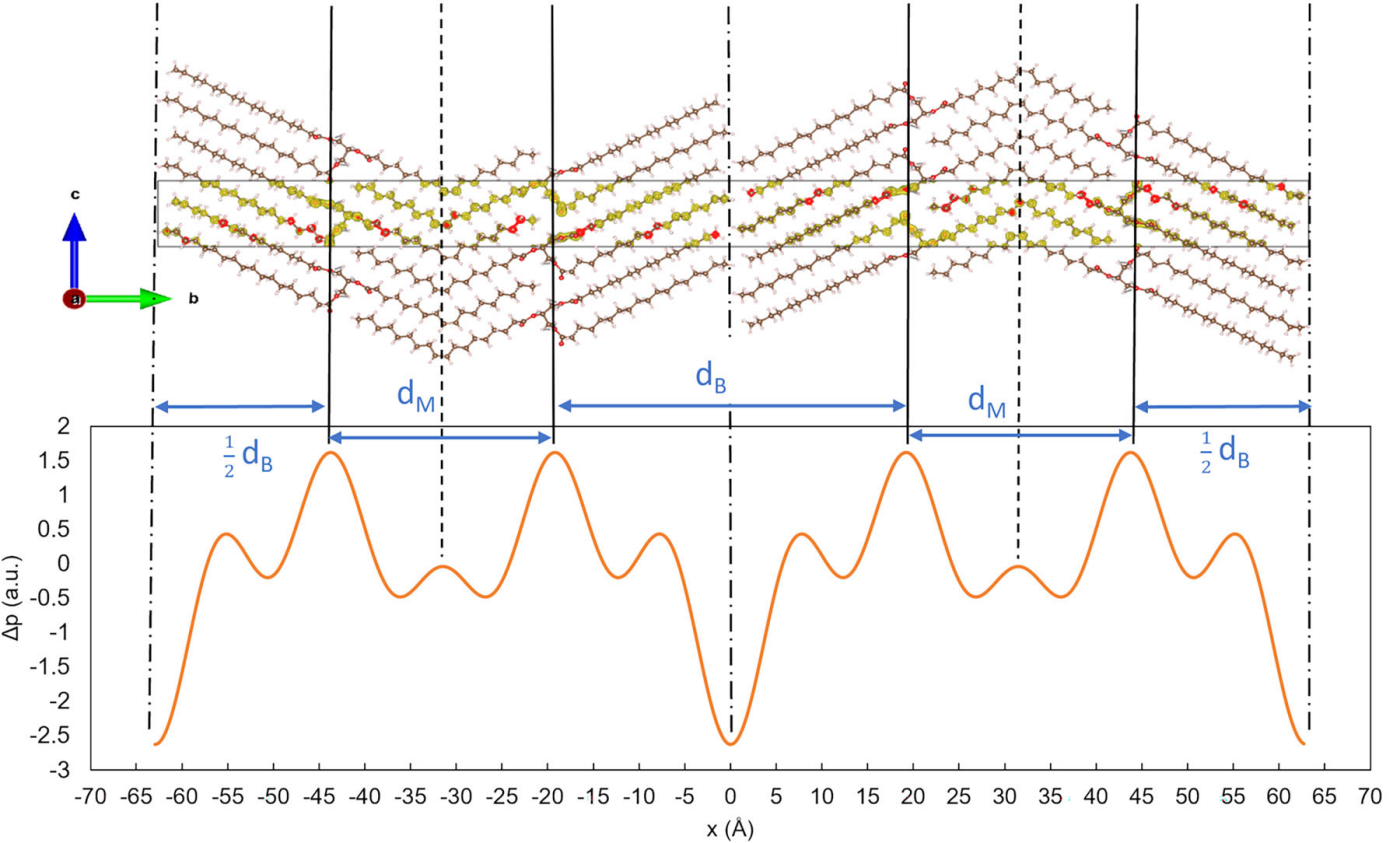
Example for the determination of the bilayer thickness (*d*_B_) from the EDP for POS in the β_1_‐3L polymorph.

The tilt angle of the hydrocarbon chains in 2L‐ and 3L‐stackings of TAGs can be estimated from electron density profiles via a geometrical approach. This requires the projected alkyl‐chain length and the actual alkyl‐chain length determined by the number of carbon atoms present. The overall thickness in a 2L stacking is given by the bilayer thickness (*d*_B_), and in the 3L stacking by the sum of the monolayer thickness (*d*_M_) and *d*_B_, such that *d* = *d*_B_ + *d*_M_, see Figure 5. The *d*_B_ can be determined from the local maximum in the EDP (Figure 6).

From the known number of carbon atoms (*N*_C_) and taking the literature of a projected bond length (C–C) along the straight hydrocarbon chains of 1.27 Å (Nagle and Tristram‐Nagle 2000), the actual alkyl‐chain length can be calculated (*d*_C_). According to this ansatz, the chain tilt angle, θ_2L_, in 2L‐phases can be approximated via (Pratama et al. 2022) (Equation 2):

(2) $$\theta_{2L}=\cos^{-1}\left(\frac{\mathrm{projected}\;d_{C}}{d_{C}}\right)=\cos^{-1}\left(\frac{\frac{1}{2}\;d_{B}-2.54\;Å}{N_{C}\;1.27\;Å}\right).$$

Note that it is necessary to account for the glycerol backbone extension in the stacking direction. This value has been refined to a value of 2.54 Å in this review, using the crystallographic data of MMM, PPP, and SSS in the β‐phase (for details see Supporting Information); similarly, we determined for 3L‐phases (Equation 3):

(3) $$\theta_{3L}=\cos^{-1}\left(\frac{\frac{1}{2}\;d_{B}-1.54\;Å}{N_{C}\;1.27\;Å}\right).$$

Note, the glycerol backbone extension in stacking direction has been refined to a value of 1.54 Å in this review, using the crystallographic data of POP, POS, and SOS in the β_1_‐ and β_2_‐phases (for details see Supporting Information). Table 6 summarizes the tilt angles estimated exemplarily for some triglycerides.

**TABLE 6:** Summary of tilt angles for SSS, POS, and BuSP in different polymorphic forms.

Triglyceride	Polymorph	Tilt angle	References
SSS	β_1_ (2L)	29.8°	van Langevelde et al. (2001b)
POS	β_1_ (3L)^1^	34.2°	van Mechelen et al. (2006a, 2006b)
	β_2_ (3L)^2^	32.5°	van Mechelen et al. (2006a, 2006b)
BuSP	α (3L)	0°	Pratama et al. (2022)
	β′ (3L)	29.7°	Pratama et al. (2022)

*Note*: For the construction of the EDPs for SSS and POS, the powder diffraction patterns were generated using VESTA on the basis of the refined crystal structures provided for SSS and POS. Data on BuSP were taken from Pratama et al. (2022). Deviations within the errors might be caused by both the uncertainty on the longitudinal glycerol backbone extension and the positional error of the EDP maxima.

**FIGURE 7:**
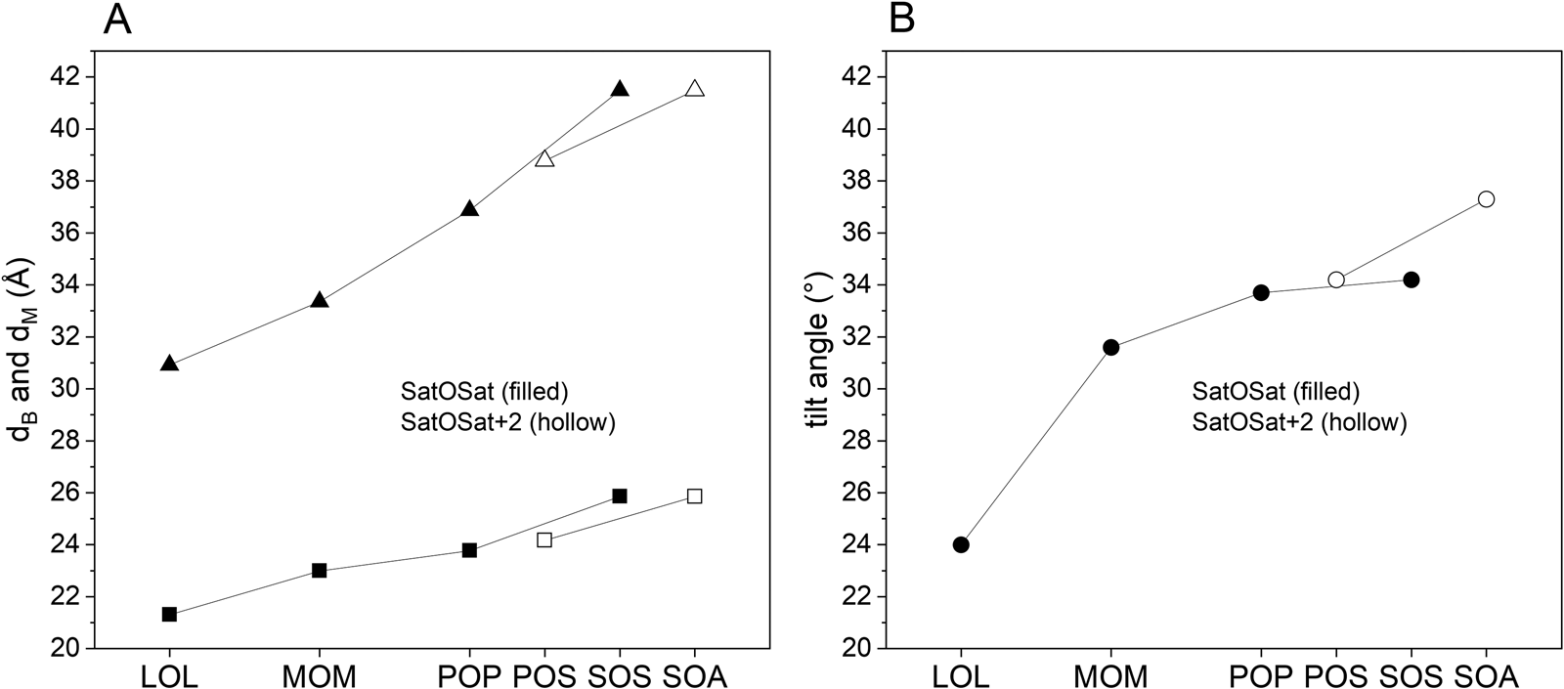
(A) Bilayer (upward facing triangles) and monolayer thickness (squares) and (B) tilt angle (circles) determined from the EPDs for LOL, MOM, POP, SOS, POS, SOA in β_1_‐3L. The associated number of carbon atoms (*N*_C_) were 12, 14, 16, 17, 18, and 19, respectively. Triglycerides of the SatOSat kind displayed as filled symbols, triglycerides of the SatOSat+2 kind as hollow symbols.

**FIGURE 8:**
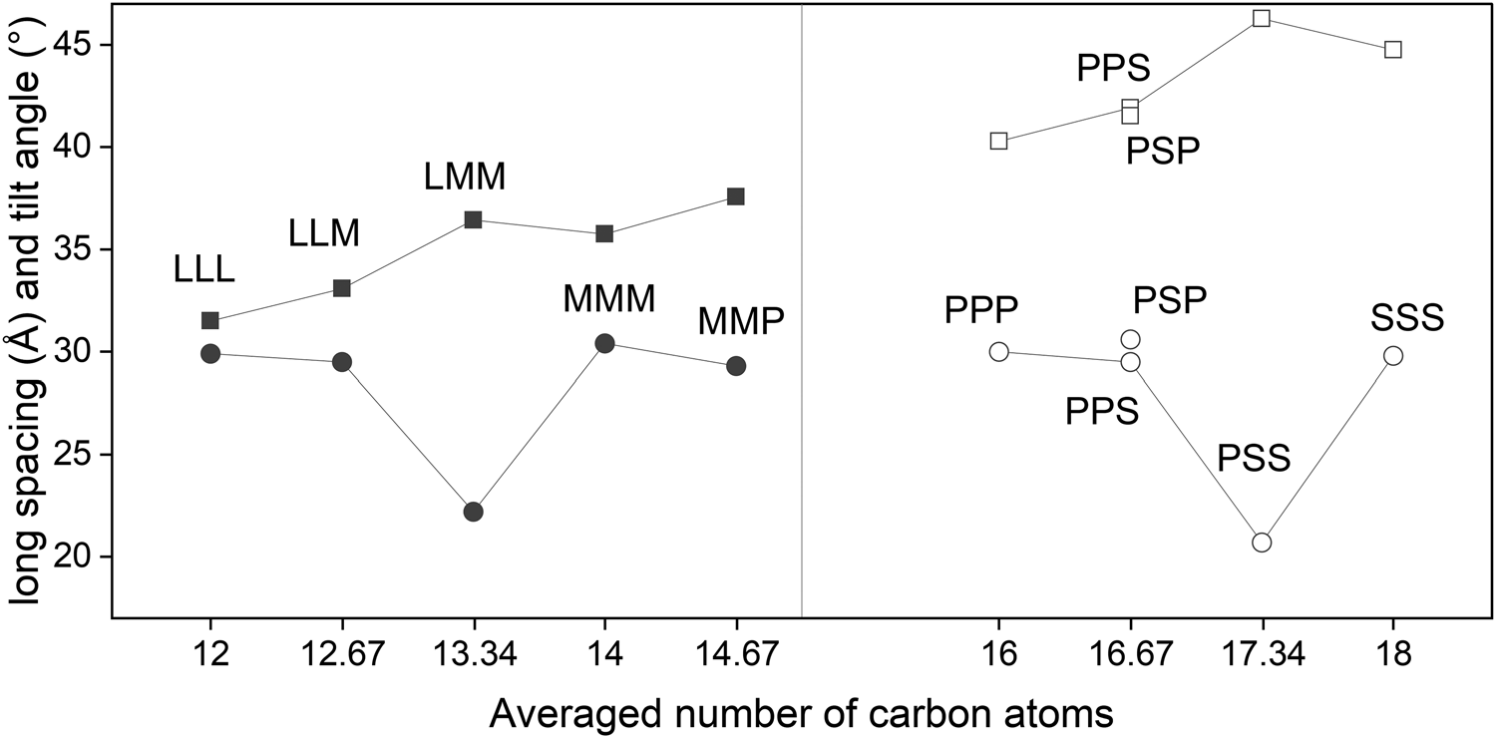
Long spacing (squares) and tilt angle (circles) plotted over the equivalent carbon numbers for LLL, LLM, LMM, MMM, MMP, PPP, PSP/PPS, PSS, and SSS. The average *N*_C_ are given as follows: 12, 12.3, 13.67, 14, 14.3, 16, 16.3, 16.67, and 18.

For BuSP, the tilt angle in the β′‐polymorph has now been corrected to 29.7°, applying the improved geometrical relationship given in Equation (3). For comparison, chain tilt angles are given for SSS, and POS in β_2_ and β_1_ phase (Table 6). The tilt angle differs depending not only on the associated projected alkyl chain length (*N*_C_), but also on the triglyceride category. For various TAGs, the bilayer thicknesses, d_B_, were used (Table 5), to determine the tilt angle shown in Figure 7 for mono‐unsaturated TAGs and in Figure 8 for saturated TAGs.

For symmetrical mono‐unsaturated TAGs (i.e., of the category SatOSat), both bilayer thickness and tilt angle increase with increasing number of carbon atoms. A similar picture emerges for asymmetrical mono‐unsaturated TAGs (i.e., of the category SatOSat+2 where the outer saturated fatty acids differ in two carbon atoms). The longer the alkyl chain, the greater the bilayer thickness and the tilt angle. Interestingly, the monolayer thickness also increases with increasing alkyl chain length, despite the monolayer being formed only of oleic‐acid chains. This clearly indicates a dominant effect on the lamellar stacking of the packing of the bilayer.

For all saturated TAGs, the long spacing (*d*) increases with increasing number of carbon atoms while the tilt angle is relatively stable. For the Sat‐2SatSat category (LMM and PSS), we can see a maximum in long spacing and a minimum in tilt angle. The inconsistencies in the long spacings and tilt angles of TAGs of the Sat‐2SatSat group might also cause demixing in binary systems.

### 4.3 Information Obtained from Peak Broadening: Crystallite Size and Strain

Physical peak broadening is caused by two aspects: the crystallite size of the powder sample and strain components (Guinebretière 2007; Spieß et al. 2009).

#### 4.3.1 Crystallite Size

The first‐order reflection in the small‐angle region allows to determining the crystallite size according to Scherrer (1918). Scherrer stated that peak width is inversely proportional to crystallite size. The crystallite size (*L*) can be calculated using Equation (4):

(4) $$L=K\frac{2\;\pi }{\beta_{\mathrm{sample}}},$$

where *β*_sample_ is the full width half maximum (FWHM) of the first‐order reflection peak on the *q*‐scale after adjustment for instrumental broadening. Note, the measured FWHM of the first‐order peak is a convolution of the instrumental width and the intrinsic sample width contribution (Equation 5):

(5) $$\mathrm{FWHM}=\beta_{\mathrm{sample}}⊗\beta_{\mathrm{instrumental}}.$$

The intrinsic sample width can be achieved by deconvolution of the measured diffraction peak with the instrumental profile or when fitting the diffraction peak with a model function by convoluting the model with the instrumental profile. In the special cases, when only Lorentzian (L) and Gaussian (G) shaped distributions are involved in Equation (5), the following equations can be applied (Z. Zhang et al. 2003):

(5a) $$\left(\mathrm{LL}\right):\;\mathrm{FWHM}=\beta_{\mathrm{sample}}+\beta_{\mathrm{instrumental}},$$

(5b) $$\left(\mathrm{GG}\right):\;\mathrm{FWH}M^{2}=\beta_{\mathrm{sample}}^{2}+\beta_{\mathrm{instrumental}}^{2},$$

(5c) $$\left(\mathrm{LG}\right):\frac{\beta_{\mathrm{sample}}}{\mathrm{FWHM}}=1+{\left(\frac{\beta_{\mathrm{instrumental}}}{\mathrm{FWHM}}\right)}^{2},$$

where FWHM, β_sample_, and β_instrumental_ are the measured width of the experimental profile, the intrinsic profile of the sample, and instrumental profile, respectively. We note that most instruments have a Gaussian‐shaped instrumental profile, while the intrinsic diffraction peak shape of the metastable polymorphs is Lorentzian, and only the β′ and β‐phases of pure TAG systems might exhibit Gaussian shaped peaks.

Further, Scherrer's equation holds a dimensionless constant *K*. In Scherrer's original work from 1918, the constant $K=2\sqrt{\ln(2)/\pi }≅0.9394$. This follows a few assumptions: (1) X‐ray waves are singly scattered on atoms (kinematical approximation) and dynamic effects are neglected. (2) the material is an “ideal powder”, that is, chaotically located identical particles, hence, crystalline size and shape distribution can be neglected. The form factor *K*_hkl_ accounts for the particle's shape. For instance, the value ∼ 0.94 describes spherically shaped crystals with inner cubic symmetry. (Vorokh 2018) The interested reader might want to study Langford and Wilson (Langford and Wilson 1978), who reviewed the Scherrer constant for various assumed platelet dimensions. When detailed shape information is missing, a value of *K* = 0.9 is a good approximation (Holzwarth and Gibson 2011). Fat crystals are more like parallelepipeds with needles and platelets as special cases, as fat crystallites grow fast laterally and slow axially, which results in thin, long, and broad platelets (Den Adel et al. 2018). We note that Scherrer's equation has been adopted to fat systems by Acevedo and Marangoni (Acevedo and Marangoni 2015). They used a value of 0.9 for the Scherrer constant in this case.

Equation (4) allows to estimate a mean of crystallize sizes up to 100 nm. In cases of crystallite sizes larger than 100 nm it is more difficult to differentiate peak broadening (FWHM), because the instrumental width is only marginally smaller in this case (Acevedo and Marangoni, 2015). It must be noted that size effects are independent of the diffraction peak order, *h*. Strain effects, however, vary or rather increase with the peak order (Den Adel et al. 2018). This means that the crystallite size determination is best performed on the first order reflection peak where the effect of strain is the smallest. It should also be noted that the crystallite size is not limited to neat, single diffraction peaks. If two phases co‐exist, and hence diffraction peaks overlap, peak fitting would allow the determination of the crystallite size of the respective phases. Finally, we would like to mention that there is an alternative way to determine the crystallite size and volume‐weighted average size distribution, which is the Fourier transformation based on Bertraut–Warren–Averbach analysis (Drits, et al. 1998; Den Adel et al. 2018; Rondou et al. 2022).

#### 4.3.2 Strain Analysis

Stain analysis is performed on imperfect crystals. Here, atoms are displaced with respect to their position in perfect crystals, which are defined to be free from any defects. These displacements result in local strains. These local strains can be significant even if the unit cell parameters are not affected (Guinebretière 2007; Spieß et al. 2009). As outlined in the analysis for crystallite size above, the diffraction peak width is utilized to determine the strain.

(6) $$\beta_{sample}^{2}cos^{2}\theta =4{\langle \varepsilon \rangle }^{2}sin^{2}\theta +\frac{K^{2}\;\lambda^{2}}{L^{2}},$$

where ⟨ε⟩ is the strain, L is the crystallite size, K is the Scherrer constant, e.g., 0.9, λ is the wavelength, θ is the incident scattering angle, β_sample_ is the FHWM adjusted by the instrumental peak broadening (see Equation 5a–c). We note that Koizumi et al. (2023) reported an instrumental peak broadening of 0.0407°, that is, Δ*q* = 2.98 × 10^−4^ nm^−1^, when accounting for the wavelength of 1.5 Å, and Penagos et al. (2024a) of 6 × 10^−3^ nm^−1^—both studies were performed using synchrotron XRD. Instrumental peak broadening is commonly greater on lab‐scale instruments.

**FIGURE 9:**
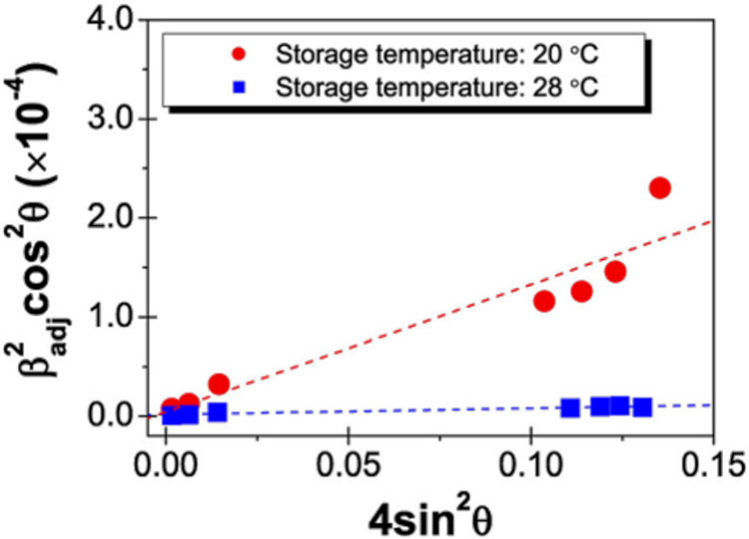
Williamson–Hall plot for cocoa butter substitute stored at different temperatures. Note: *β*_adj_ refers to our *β*_sample_ nomenclature. The figure is reproduced from Koizumi et al. (2023) with permission from the Royal Society of Chemistry.

The strain accumulated in crystals can be determined when plotting β_sample_^2^ cos^2^θ versus 4 sin^2^θ—which is known as the Williamson‐Hall plot (Guinebretière 2007; Koizumi et al. 2023; Spieß et al. 2009). The slope represents the strain, and the intercept represents the size of the crystallites. Figure 9 gives the Williamson‐Hall plot for a cocoa butter substitute stored at different temperatures. The accumulated strain in cocoa butter substitute (CBS) is stated to promote the phase transitions from β′ into β and cause fat bloom of the CBS (Koizumi et al. 2023, 2022).

Recently, Stobbs et al. (2024, 2025) developed a modified William–Hall plot allowing determination of both domain sizes and microstrain in cocoa butter. This refined method is especially useful when investigating the effect of lattice strain on crystal growth and the stability of fat crystal networks.

## 5 Nanostructural Packing Information Deduced from WAXS

Besides the unit cell introduced above, the 3D‐subcell characterizes the fatty acid packing and is used to distinguish between different polymorphic forms of TAGs. The 3D‐subcell structure refers to the cross‐sectional packing of the zigzag hydrocarbon chains, and hence, is a local property of the crystal.

### 5.1 The Concept of the 3D‐Subcell of Chain Packing

In the lipid crystals, the alkyl chains can adopt a variety of different modes of lateral packing, which are described by subcells. The concept of subcells was first described by Vand (1951) and later applied to trilaurin (LLL) by Vand and Bell (1951). Briefly, the subcell concept states that if a crystal is composed of periodic structures, a smaller unit than the unit cell can be used to simplify the structure determination. In TAGs and similar molecules (e.g., fatty acids), the alkyl chains pose a periodic structure. Here, the alkyl chains form a three‐dimensional array due to their translation between equivalent positions within periodic carbon chains and adjacent chains. Just as the unit cell, the subcell has three dimensions, a_s_, b_s_, and c_s_. The hydrocarbon chains lie along the c_s_ axis of the subcell, forming a plane parallel to the b_s_ axis. Since there are only two CH_2_ groups per subcell, c_s_ can be fixed to approximately 2.54 Å (Small 1984).

**FIGURE 10:**
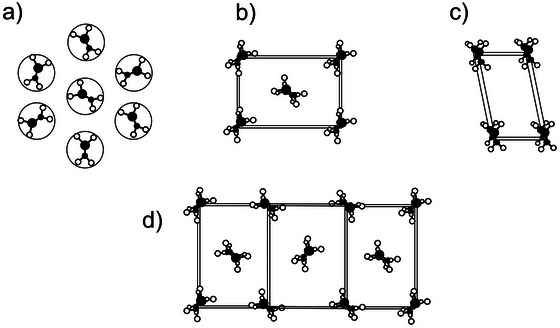
Schematic depiction of common 3D‐subcells (explanation in text) in triglycerides. (a) Hexagonal, (b) orthorhombic, (c) triclinic parallel, and (d) hybrid 3D‐subcells.

The subcells with triclinic, orthorhombic, monoclinic, and hexagonal symmetry are defined, see Figure 10a–c. Further, the alkyl planes arrange in either a parallel or perpendicular fashion with respect to their neighbors. The exception is the hexagonal packing (Figure 10a) without a specific arrangement of the hydrocarbon chains, as they are freely rotating. Note that for this reason, the hydrocarbons are not tilted in the α‐phase. Complicating the matter further, the subcell itself can be simple or hybrid (Hernqvist 1988), of which the latter is defined to be those involving more than two different asymmetric units. A TAG assuming a hybrid subcell was first observed by Sato et al. (2001), for PPM in β′‐2L displayed in Figure 10d.

The unit cell geometry can be different from the subcell; for example, a monoclinic crystal system may consist of an orthorhombic perpendicular subcell or a triclinic parallel subcell (Sato 2018b). The β′‐polymorph of PPM was found to have a monoclinic unit cell despite having an orthorhombic sub‐cell (Sato et al. 2001). Other examples are the β‐polymorphs of POP and SOS, both mono‐unsaturated TAGs, reported to have a monoclinic unit cell contrary to the triclinic subcell (van Mechelen et al. 2006a). Last, the unit cells and 3D‐subcells can also be the same, as for the β′‐polymorph of C_n_C_n+2_C_n_‐type TAGs (*n* = even) such as PSP (van Langevelde et al. 2000).

With c_s_ translation being 2.54 Å, the 3D‐subcell is merely a cut through the chains parallel to the methyl end plane and glycerol plane, that is, the a_s_‐b_s_‐plane. The a_s_‐b_s_‐plane then describes the 2D subcell. There are different 2D rectangular lattices, including orthorhombic perpendicular (O_⊥_) and orthorhombic parallel (O_//_). There are also oblique lattices including triclinic parallel (T_//_) and monoclinic parallel (M_//_) (Small 1984). It is worth mentioning that the 2D subcell lattices, hexagonal, rectangular, and oblique, are universal for the given polymorphs of fats and are used to calculate the area per hydrocarbon chain (see Section 5.2). In the following, the more common 3D subcells are used to distinguish polymorphic forms.

The three most common polymorphic forms are α, β′, and β. Accordingly, the α‐polymorph has a hexagonal subcell (H), which consists of freely rotating hydrocarbon chains. The chains do not tilt with respect to the layer normal. The β′‐polymorph is most commonly assigned an orthorhombic perpendicular subcell (O⊥) in which adjacent hydrocarbon chains are orientated in an orthogonal fashion, see Figure 10b. Finally, a triclinic parallel subcell (T_//_) with parallel‐orientated chains is commonly assigned to the β‐polymorph, see Figure 10a–c, respectively. The chains are inclined by approximately 30°–33° (see Section 4.2), whereby the angle of inclination in the β‐polymorph is greater than the respective angle in the β′‐polymorph (Garti and Sato 1988; Himawan et al. 2006; Wesdorp et al. 2013). Note that generally a denser chain packing is accompanied by a greater chain tilt.

A summary of short spacings reported for various TAGs in the three main polymorphic forms is given in Table 7. For unsaturated TAGs such as POP and SOS, a sub‐α form was found, often denoted as γ, and, most recently, for POS and SSS, a more stable β phases were found (Ghazani and Marangoni, 2018, 2023). The polymorphic structure of the γ‐phase is quite unique. Although the melting temperature is below that of the α‐polymorph, the subcell packing is similar to that of the β′‐polymorph, exhibiting an orthorhombic subcell. Yet, the dimensions (associated *d*‐spacings) are different, see Table 7. For γ, a characteristic reflection at 4.7–4.8 Å has been reported for POP and SOS (Bayés‐García et al. 2013b; Lutton 1955b; Sato et al. 1989).

**TABLE 7:** Short spacings of triglycerides in various polymorphs reported in the literature.

TAG	α	γ	β′	β	References
Mono‐acid saturated TAGs					
SSS	4.15		4.2, 3.8	4.6, 3.9, 3.7	Oh et al. (2002)
	4.1–4.2		4.2, 3.8	4.6, 3.7, 3.85	Lavigne et al. (1993)
				β_1_: 4.57, 3.84, 3.68 β_2_: 4.61, 3.86, 3.70	Ghazani and Marangoni (2023)
PPP	4.18		4.22, 3.89	β_1_:4.62, 3.9, 3.79	Bhaggan et al. (2018a, 2018b)
	4.15			β_2_: 4.6, 3.85, 3.7^a^	Kellens et al. (1990b); Sato et al. (1999)
	4.1		4.3, 4.2, 3.8	4.6, 3.8, 3.7	Macridachis et al. (2022)
	4.2		4.2, 3.8	4.6, 3.9, 3.8	Takeuchi et al. (2003)
MMM	4.2		4.2, 3.8	4.6, 3.9, 3.8	Takeuchi et al. (2003)
LLL	4.13		4.23, 3.90	β_1_: 5.35, 5.22, 4.57, 4.41, 3.90, 3.82 β_2_: 5.37, 5.23, 4.62, 4.57, 4.42, 3.89, 3.78	Yoshikawa et al. (2022)
	4.2		4.2, 3.8	4.6, 3.9, 3.8	Takeuchi et al. (2003)
Mixed‐acid saturated TAGs					
PSS	4.18		4.22, 3.88		Bhaggan et al. (2018a, 2018b)
	4.11		4.19, 3.81	4.52, 3.83, 3.65	Bouzidi and Narine (2012)
PSP	4.13		4.33, 4.2, 4.03, 3.83		Bhaggan et al. (2018a, 2018b)
PPL	4.14		β′_1_: 4.39, 4.23, 4.03, 3.83 β′_2_: 4.25, 3.84	5.47, 4.71, 4.57, 3.90, 3.74	Kodali et al. (1990)
PPM	4.13		β′_1_: 4.36, 4.19, 3.99, 3.80 β′_2_: 4.21, 3.80		Kodali et al. (1990)
SLL	4.13		β′‐2L: 4.23, 4.06, 3.77 β′‐3L: 4.30, 4.17, 3.94	4.6, 3.84	Lutton (1948)
LLM	4.15		4.32, 3.84	4.6, 4.55, 3.84, 3.76	Danthine (2024)
MML	4.14		β′_1_: 4.43, 4.26, 4.05, 3.83 β′_2_: 4.22, 3.86		Danthine (2024)
Short‐chain saturated TAGs					
BuSP	4.1		4.33, 4.14, 3.80 (3L)		Pratama et al. (2022)
Trans‐unsaturated TAGs					
SES	4.2			5.3, 4.6, 3.9, 3.6	Elisabettini et al. (1998)
	4.2			4.6, 3.9, 3.8	Elisabettini et al. (1998)
ESS	4.1		4.4, 4.3, 4.1, 3.9	5.3, 4.6, 3.9, 3.7	Elisabettini et al. (1998)
PEE	4.2		4.2, 4.1, 3.9		Elisabettini et al. (1998)
EPP	4.2		4.4, 4.3, 4.2, 3.9		Elisabettini et al. (1998)
PEP	4.2		4.4, 4.2, 4.1, 3.9		Elisabettini et al. (1998)
Unsaturated TAGs					
OPO	4.2		4.3, 4.0	4.8, 3.8, 3.7	Bayés‐García et al. (2015)
OSO	4.05		4.12, 3.85	5.28, 4.52, 3.77	Kodali et al. 1987
POO	4.1		β′_2_: 4.3, 4.1 (3L)		Bayés‐García et al. (2016)
SOO	4.1		β′_2_: 4.3, 4.1 (3L)		Bayés‐García et al. (2016)
OOS	4.2		4.6, 4.4, 4.0		Zhang et al. (2009)
POL	4.1		β′_2_: 4.2, 3.8		Bayés‐García et al. (2016)
SSO	4.15, 3.75^b^		4.04, 3.74, 4.64, 4.4		Lavery (1958)
PPO	4.2		4.6, 4.4, 4.2, 4.0, 3.8		Bayés‐García et al. (2015)
	4.1		β′2: 4.2, 3.9 (2L) β′1: 4.2, 3.9 (3L)		Macridachis et al. (2022)
POP	4.2	4.8, 4.7, 4.5, 4.0, 3.9	β′_2_: 4.3 4.2 3.9 (2L) β′_1_: 4.3, 4.0 (2L)	β_2_: 4.6, 4.1, 3.8, 3.7	Bayés‐García et al. (2013b)
POS				β_3_: 4.61, 3.98, 3.87, 3.75, 3.66 β_2_: 4.6, 3.97, 3.85, 3.74, 3.69, 3.65 β_1_: 4.6, 4.04, 3.93, 3.86, 3.7	Ghazani and Marangoni (2018)
			β′: 5.56, 4.56, 4.36, 4.11, 3.84, 3.63		Lutton (1951)
SOS	4.1			4.6	Zhang et al. (2009)
	4.3, 4.1, 4.2^c^		4.2, 3.8	4.6, 4.0, 3.9, 3.8, 3.7	Ueno et al. (1997)
	4.21		4.24, 3.9	4.58, 3.65	Takeuchi et al. (2002a)
	4.21	4.72, 4.50, 3.88, 3.63	4.30, 4.15, 4.02, 3.95, 3.83, 3.70	β_2_: 4.58, 4.00, 3.90, 3.75, 3.67, 3.57 β_1_: 4.58, 4.02, 3.97, 3.85, 3.80, 3.65	Sato et al. (1989)
	4.2		4.18‐4.24, 3.67‐3.7	4.58, 3.81	Baker et al. (2014)
SLS	4.12	4.74, 4.50, 3.81			Lutton (1955b)

*Note*: Units are given in Ångström.

^a^ β_1_ and β_2_ differ the intensity of the reflections, β_1_ shows reflections of higher intensities then β_2._

^b^ both short spacings describe a sub‐α‐3L.

^c^ The three short spacings for the α assume also three different long spacings: 53, 44, and 50 Å, respectively.

Polymorphs of similar subcell, e.g., β_1_ and β_2_ (from V and VI)—not to be confused with β‐2L and β‐3L, with the latter denoting the lateral packing in either double or triple stacking—differ in thermodynamic stability. The melting point of β_1_ is greater than that of β_2_ phase. The peak positions oftentimes only differ slightly and differ only in signal intensity. Different β phases are a common observation and have been found for various mono‐acid TAGs (Ghazani and Marangoni 2018, 2023; Takeguchi et al. 2020).

For mono‐unsaturated TAGs, assuming the 3L stacking, a difference in the subcell packing was discussed (Engström 1992). This was confirmed by FT‐IR analyses for symmetrical mono‐unsaturated TAGs (Garti 2001). Accordingly, the subcell packing of the saturated leaflet and oleoyl leaflet assumes different chain orientations. For example, in the γ‐polymorph of SOS, POP, SRS (R: ricinoleic acid), and SLiS (Li: linoleic acid), the saturated leaflets were found to be arranged in a parallel fashion, whereas the oleic, ricinoleic, and linoleic chains were freely rotating in a hexagonal subcell. Also, in the β′‐phase, the oleoyl leaflet was found to remain in the hexagonal subcell, whereas the saturated leaflet was orthorhombic perpendicular. For SOS and POP, two β‐phases, β_1_ and β_2_ were found. The less stable β_2_ phase showed differences in the subcells per leaflet. The saturated leaflets were found to be triclinic parallel, whereas the oleoyl leaflets were either also triclinic parallel or orthorhombic parallel. All differences were diminished in the most stable polymorphic form, being β_1_, with all leaflets assuming the triclinic parallel subcell (3D). According to Engström (1992), different subcells were also identified for molecular compounds of SOS/OSO and others. Here, the saturated chains and unsaturated chains each pack in different leaflets, assuming different subcells, triclinic and orthorhombic, respectively. This alludes to the fact that assigning only a polymorphic form in systems containing unsaturated TAGs via the subcell spacings is inadequate. Or in other words, when talking about the most common polymorphic forms, α, β′, and β, and their respective hexagonal, orthorhombic, and triclinic sub cells refer strictly to the bilayer region.

The strongest reflection observed for a β‐phase varies between 4.5–4.6 Å. A systematic study on the β‐2L form of saturated and trans mono‐unsaturated TAGs by van Mechelen et al. (2008b) revealed a pair of strong diffraction maxima at 4.6 Å. Rare in this context, the Miller indices (110) and (111) could be assigned, characteristic of the triclinic β‐2L structure. In contrast, the β‐3L phases of mono‐unsaturated TAGs, such as POP and SOS, show only the (111) reflection around 4.6 Å in the WAXS region (van Mechelen et al. 2006a, 2006b). While only a few research papers documented the Miller indices to the reported short spacings, van Mechelen et al. compared the two strongest reflections around 4.6 Å with the c_s_ dimension, which corresponds to the TAG chain lengths of the mixed‐acid saturated TAGs. The (110) was found to vary with the chain length (i.e., c‐axis), decreasing with an increasing chain length; the (111) reflection remained constant over a wide c‐axis range, comparing LLL and SSS. As the differences for (110) are small between 4.58–4.60 Å, it seems fair to conclude that the fatty acid composition within one TAG category (i.e., mono‐acid saturated, mixed‐acid saturated, mono‐unsaturated TAGs) has no notable effect. However, across these TAG categories, differences can be seen. The WAXS region diffraction pattern differs with the fatty acid composition of the TAG molecules. Further, the fatty acid composition determines if a TAG assumes the 2L or 3L stacking, e.g., SOS assumes the 3L stacking in β′ whereas POP remains in 2L (Sato et al. 1989).

In summary, TAGs occur in multiple polymorphs, not limited to the ones listed in Table 7, adding to the complexity of this research field. The most prominent example is cocoa butter, a mixture of mainly POS, SOS, and POP forming a mixed crystal with six polymorphic forms (Wille and Lutton, 1966). The occurrence of specific polymorphic forms also depends on the TAG composition of a fat. For example, Simone et al. (2024) reported different polymorphic pathways for different cocoa butter equivalents of varying amounts of SOS, POP, and POS. The SOS‐rich samples crystallized in 3L‐phases, namely, γ‐3L, the β′‐3L, and the β‐3L, whereas the POP‐rich samples crystallized first in α‐2L and β′‐2L and took months to transition into β‐3L.

Lastly, the WAXS region can be used to determine the relative amount of a crystalline phase in a specific polymorphic form to monitor emerging and vanishing phases and the distribution thereof. The difficulty arises when dealing with transient systems undergoing phase transition, where an assignment of reflections/peaks to a polymorphic form might not be fully justified. Both solid fat content determination and general difficulties encountered in the analysis of phase transitions in more complex fat mixtures are discussed in Sections 6 and 7.

### 5.2 Area per Chain Determination

**FIGURE 11:**
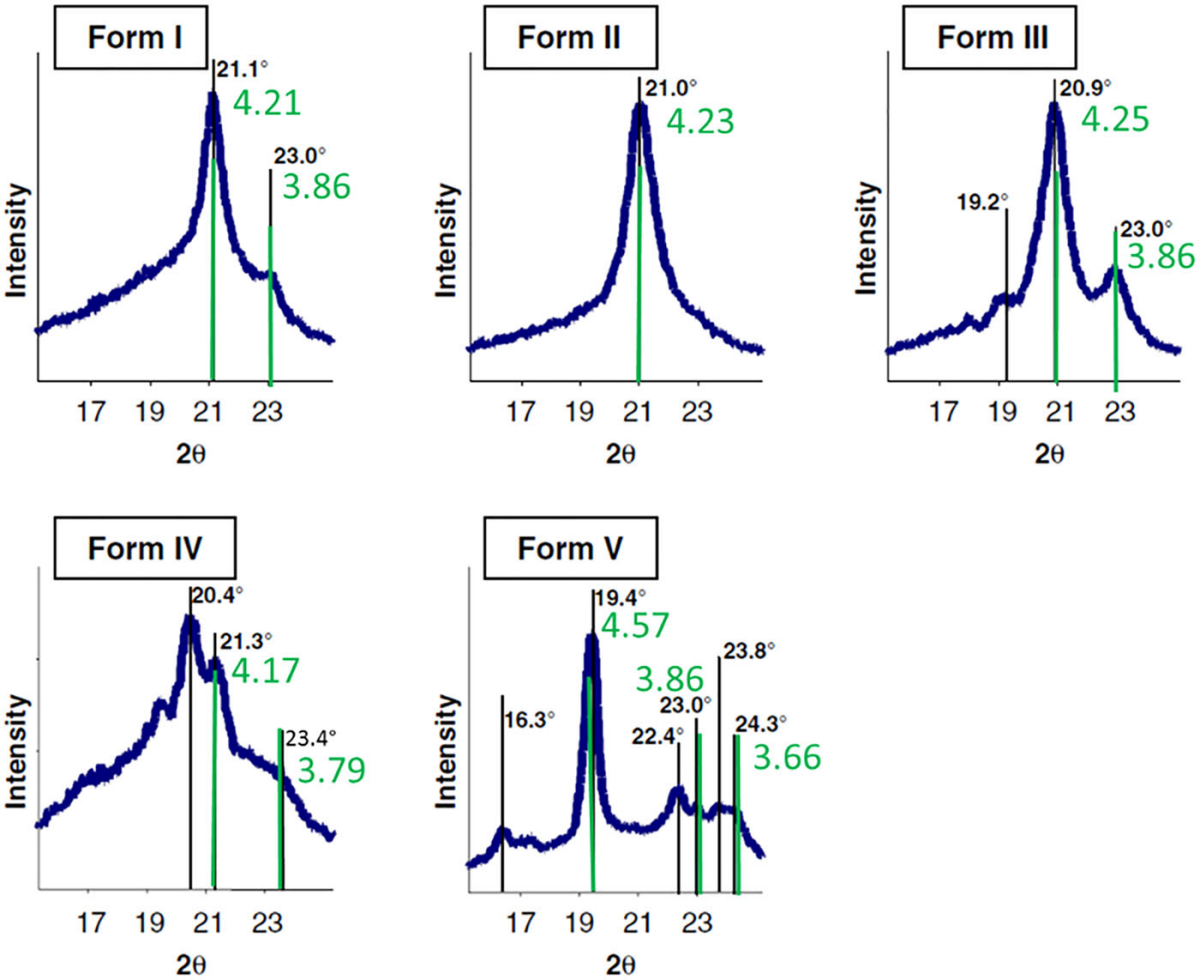
WAXD examples of polymorphs I to V of cocoa butter. Green bars and lattice spacings in Å have been added displaying the *d*_10_‐repeat (form II: hexagonal packing), the *d*_11_ > *d*_20_ repeats for the form I, form III and form IV (rectangular packing with four nearest neighbors) and the three lattice spacings defining the oblique packing of chains in the form V (form I = γ‐phase, form II = α‐phase, form III = β_2_’‐phase, form IV = β_1_’‐phase, and form V = β_2_ phase). *Source*: The figure adapted from Pore et al. (2009) with permission from Wiley.

As early as 1950, the 3D‐subcell information and 2D cross‐area of chains were discussed (Abrahamsson et al. 1978; Lutton 1950; Small 1984; Vand 1951). The cross‐section of the α‐polymorph is hexagonal and a low‐density structure with an area (cross‐section) per chain of about 20 Å^2^. It universally involves untilted or perpendicular chains with respect to the layer surface. Both β′ and β are denser in cross‐section packing than α with the area per chain of about 18.5 Å^2^ (Lutton, 1950). Simplified 2D subcells are employed for the determination of the area per chain, A_C_. The 3D‐hexagonal subcell in the α‐phase can be simplified to a hexagonal unit cell, and the 3D orthorhombic subcell in the β′‐phase can be simplified to a rectangular cell (Figure 11). The area per chain with “hexagonal packing” can be calculated as Equation (7) (Small 1984):

(7) $$A_{C}=2\frac{{d_{10}}^{2}}{\sqrt{3}}\;,$$

where *d*_10_ is the *d*‐spacing of the only diffraction peak recorded at about *q*_10_ = 1.53 Å^−1^.

The area per chain in with “rectangular packing” can be calculated as Equation (8) (Marsh 2012):

(8) $$A_{C}=\frac{d_{20\;}d_{11}}{\sqrt{1-{\left(\frac{d_{11}}{2\;d_{20}}\right)}^{2}}},$$

where *d*_20_ is the corresponding *d*‐spacing for the medium‐intense peak at about *q*_20_ = 1.64 Å^−1^ and d_11_ is the corresponding *d*‐spacing of the strong peak at about *q*_11_ = 1.51 Å^−1^.

**FIGURE 12:**
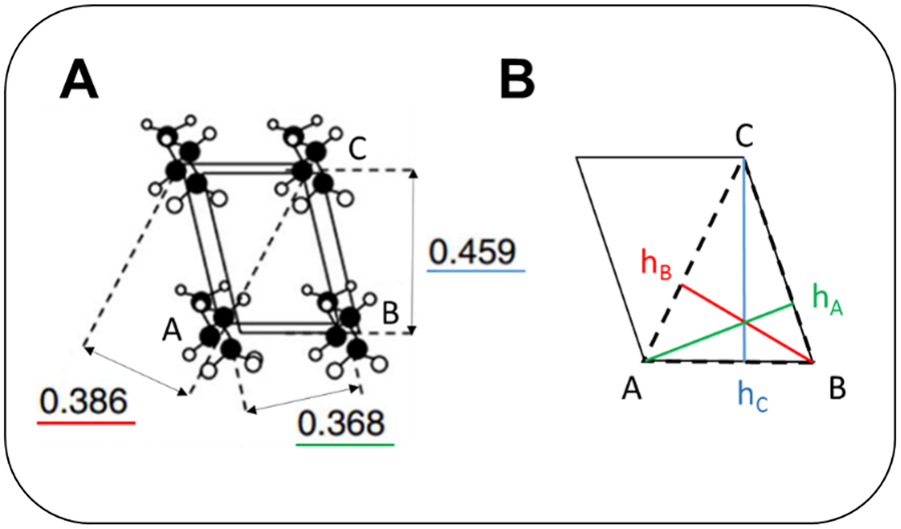
(a) Demonstration of the oblique packing of chains in the form V. (b) Half the subcell is displaying a triangle with the given heights *h*_A_, *h*_B_, and *h*_C_. These heights refer to the *d*_10_, *d*_−11_, and *d*_01_‐spacings of the oblique subcell or to *d*_0 16 2_, *d*_1 17 −1_, and *d*_101_ in the triclinic unit cell (van Mechelen et al. 2007).

The simplified subcell of the β‐phase reflects oblique packing (see parallelogram in Figure 12b), which can further be divided into two triangles determined by the three given heights. Hence, the area per chain in with “oblique packing” of chains can be calculated using Equation (9):

(9) $$A_{C}=\frac{2\cdot {h_{A}}^{2}{h_{B}}^{2}{h_{C}}^{2}}{\sqrt{\left(h_{A}h_{B}+h_{B}h_{C}+h_{C}h_{A}\right)\left(-h_{A}h_{B}+h_{B}h_{C}+h_{C}h_{A}\right)\left(h_{A}h_{B}-h_{B}h_{C}+h_{C}h_{A}\right)\left(h_{A}h_{B}+h_{B}h_{C}-h_{C}h_{A}\right)}}.$$

The heights of the triangle (see Figure 12b) can be replaced by *h*_A_ = *d*_10_ < *h*_B_ = *d*_−11_ < *h*_C_ = *d*_01_. Then follows (Equation 10):

(10) $$A_{C}=\frac{2\cdot {d_{10}}^{2}{d_{-11}}^{2}{d_{01}}^{2}}{\sqrt{\left(d_{10}d_{-11}+d_{-11}d_{01}+d_{01}d_{10}\right)\left(-d_{10}d_{-11}+d_{-11}d_{01}+d_{01}d_{10}\right)\left(d_{10}d_{-11}-d_{-11}d_{01}+d_{01}d_{10}\right)\left(d_{10}d_{-11}+d_{-11}d_{01}-d_{01}d_{10}\right)}}.$$

Note, the determination of the area of a triangle by knowing only its three heights is solved by applying Heron's formula (Mitchell 2005). Note, the area of the triangle in Figure 12b is half the area of *A_C_*.

Table 8 summarizes the *A_C_* determined from WAXS information for SSS, POS, and BuSP. From the BuSP data, it can be seen that the area per chain decreases from α to β′. In comparison, the *A_C_* of SSS in β is even lower than the *A_C_* of BuSP in β′. POS gives multiple reflections in the WAXS region, more than the typical quadruplet in cocoa butter. Here, the reflections of corresponding indices were used for the determination. The difference between β_1_ and β_2_ is negligible.

**TABLE 8:** Summary of area per chain determination WAXS information for selected triglycerides in different polymorphic forms.

Triglyceride	Polymorph	Area per chain A_C_ (Å^2^)
SSS	β_1_	18.8
POS	β_1_	18.9
	β_2_	18.9
BuSP	α^3^	19.4
	β′	18.9

*Note*: *A_C_* calculated from powder diffraction patterns generated using VESTA (Momma and Izumi 2011) and crystal structure data for SSS from van Langevelde et al. (2001b) and POS from van Mechelen et al. (2006a, 2006b). Data for BuSP were taken from Pratama et al. (2022).

It was further reported that the cross‐sectional area (i.e., *A_C_*) increases with temperature due to an increase in chain mobility. (Akita et al. 2005)

## 6 Solid Fat Content Determination from the WAXS

X‐ray diffraction is also a useful tool to determine the solid fat content (SFC). Two methods are presented. The first is model‐free and is based on the determination of the total diffraction intensity after correct subtraction of the diffuse scattering contribution (fluid fraction). The other relies on model fitting procedures, also allowing for computation of the solid fraction per polymorphic form.

### 6.1 Model‐Free SFC Determination from WAXS Data

In this method, the recorded scattering data is transformed to diffraction data by removing the fluid scattering contribution from each respective SAXS/WAXS pattern. The fluid content in each scattering pattern is different, that is, displaying a specific fluid fat fraction, *f*, with respect to the pure fluid phase. The scaling factor is determined using Equation (11):

(11) $$f\left(t\right)=\frac{\sum_{q=9.4}^{10.2}I_{\mathrm{sample}}\;\left(t\right)}{\sum_{q=9.4}^{10.2}I_{\mathrm{fluid}}\;\left(t_{0}\right)},$$

with $I_{\mathrm{sample}}\left(t\right)$ being the intensity of any acquired scattering pattern and $I_{\mathrm{fluid}}\left(t_{0}\right)$ denoting the intensity obtained for the 100% fluid sample (*t*_0_ = 0). For this normalisation step, only intensity data displaying solely diffuse scattering should be used, for example, for *q* between 9.4 and 10.2 nm^−1^.

**FIGURE 13:**
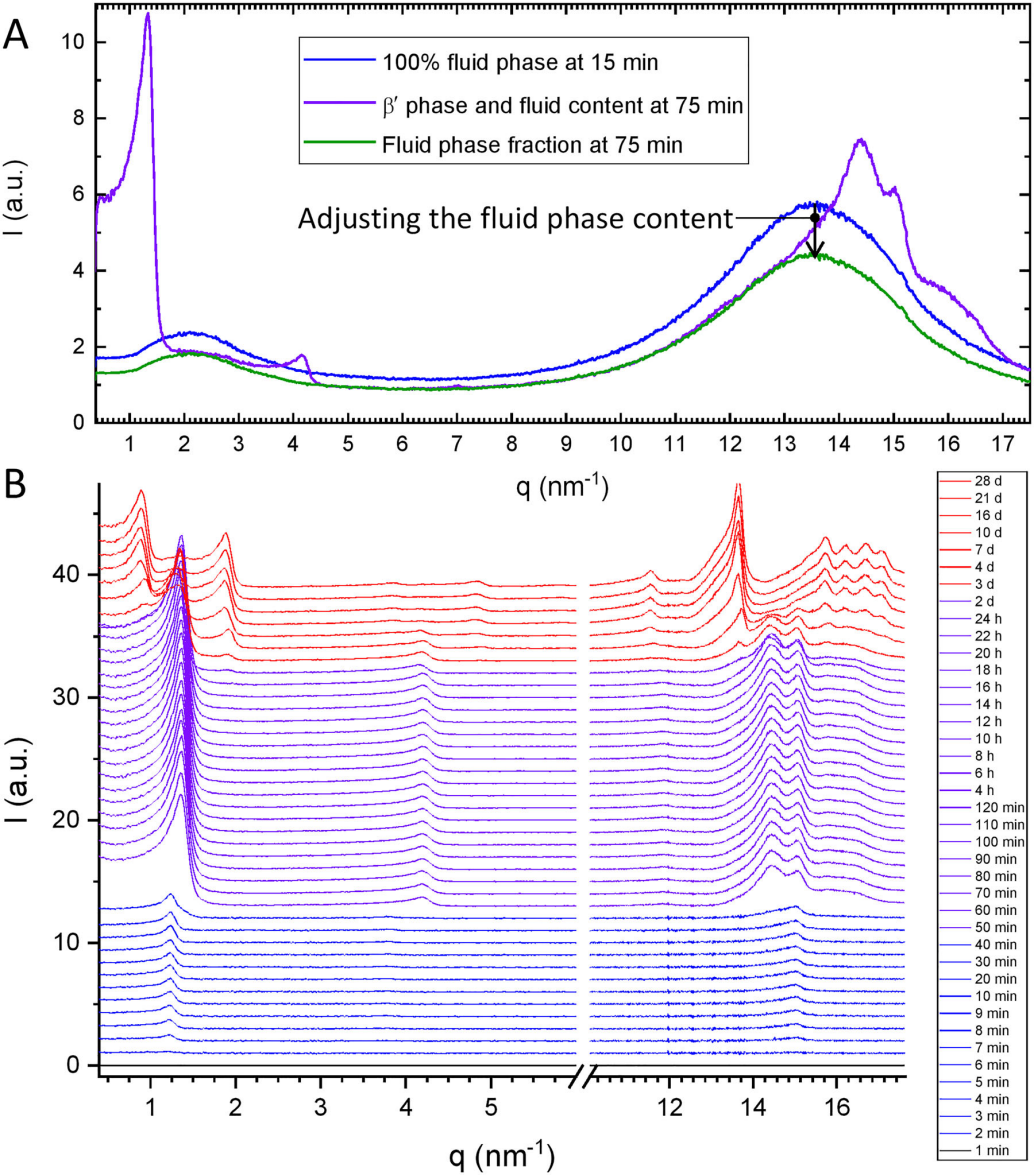
Crystallisation of CB during isothermal hold at 20°C during the period of one month. (a) SAXS and WAXS data for cocoa butter (purple solid line) after crystallizing isothermally for 70 min, pattern of fluid phase after 1 min hold (blue line), and pattern of fluid phase after rescaling to match the CB pattern at 70 min (green line) are shown. (b) SAXD/WAXD pattern of CB after subtracting the fluid scattering contribution. Note, the diffraction peaks, particularly in the SAXD regime, are smeared toward low *q* due to having employed a line‐focus camera in this case.

The corrected sample signal displaying the diffraction data is then given as Equation (12):

(12) $$I_{\mathrm{cryst}}\left(q\right)=I_{\mathrm{sample}}\left(q\right)-f\cdot I_{\mathrm{fluid}}\left(q\right),$$

with *I*_cryst_(q) being the diffraction pattern after subtraction of its fluid phase contribution. Figure 13a demonstrates how the fluid phase content is subtracted for the pattern taken after 70 min of isothermal hold. An overview of the diffraction data acquired of cocoa butter during isothermal crystallization at 20°C is shown in Figure 13b.

Using the WAXD data, one first determines the degree of crystallinity (DoC) as follows:

1. After subtraction, the fluid contribution, the total intensity of the WAXD data is determined from *q* = 12–17.5 nm^−1^, defining the solid fat contribution of each measurement ($\sum_{q=12}^{q=17.5}\mathrm{WAX}D_{i}\left(q\right)$).
2. The fluid contribution is given by the scaling factor, *f*_i_ (Equation 11). Note that the scaling fraction runs between 0 and 1, which is multiplied by the total intensity of the WAXS data of the pure fluid phase determined from *q* = 12–17.5 nm^−1^. This value defines the fluid fat contribution $\left(f_{i}\cdot \sum_{q=12}^{q=17.5}\mathrm{WAXS}\left(q\right)\right)$.
3. The DoC of each measurement, *i*, is then calculated by using Equation (13):
(13) $$\mathrm{Do}C_{i}=\frac{\sum_{q=12}^{q=17.5}\mathrm{WAX}D_{i}\left(q\right)}{\sum_{q=12}^{q=17.5}\mathrm{WAX}D_{i}\left(q\right)+f_{i}\sum_{q=12}^{q=17.5}\mathrm{WAXS}\left(q\right)}.$$

**FIGURE 14:**
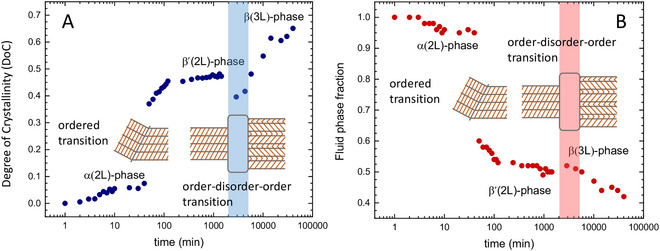
Degree of Crystallinity (a) and fluid phase fraction (b) over time obtained from WAXD data for cocoa butter (data refer to the experiment shown in Figure 13). Noteworthy, the α‐2L to β′‐2L transition displays no disorder, while the β′‐2L to β‐3L transition is an order–disorder–order transition (see schemes).

The DoC and the fraction of the fluid phase determined for each time frame are depicted in Figure 14. At first, the DoC increases in a first step, marking the formation of a α‐2L phase (DoC plateau at 6%). A greater increase in DoC is accompanied by the transition into a β′‐2L phase (DoC = 45%). We interpret this first polymorphic transition from the α to β′ phase as an ordered transition, which is characterized by a diffusionless, highly cooperative phase transformation (Rappolt et al. 2008). Here, neighboring α and β′ lamellar stacks are inclined at their interface such that their lattices match perfectly. That is, at the interface, the orientation of the hydrocarbon chains is the same in the α and β′ phase. In contrast, when the β‐3L phase is formed, the DoC drops slightly, which is characteristic of an order–disorder–order transition. Since this 3L‐stacking is reached by local phase separations of saturated and mono‐unsaturated hydrocarbon chains until new bilayers and monolayers are formed, the β′‐2L to β‐3L transition involves the diffusion of hydrocarbon chains and concomitantly the induction of disordered regions as observed in the intermittent drop of the DoC.

Importantly, due care must be taken when interpreting the DoC values deduced from WAXS with respect to SFC values reported from NMR. Strictly speaking, DoC values are not identical to the solid fractions by weight but instead refer to the crystalline solid volume fraction within the material. In our example, the DoC determined by WAXS relates to the SFC by response factors of 2, 1.32, and 1.25 with reference to the α, β′, and β phase regions in the DoC curve of Figure 14. Note that these empirical correction factors were determined by comparison with NMR‐determined SFC values on cocoa butter published by Müller and Careglio (2018). The lower DoC values are mainly explained by undetected amounts of short‐range assemblies of TAGs being in their solid state, for example, found in defect zones within the crystal or at the interfaces of the crystals with the fluid phase. In other words, the amount of diffusively scattering material, hosting fatty acids in all‐trans conformation, is surprisingly high, but clearly diminishes toward the formation of the stable β phase.

### 6.2 SFC Deduced from Model Fitting of the WAXS Data

**FIGURE 15:**
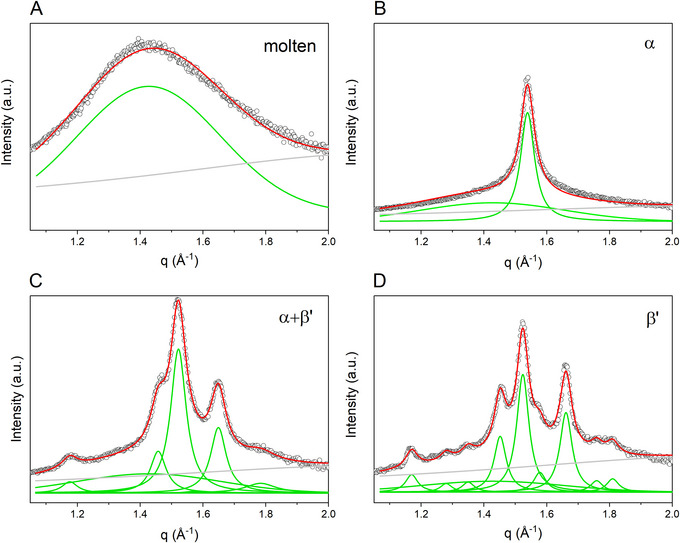
WAXS analysis for BuSP. (a) At 60°C in the sample is fully in the molten state. (b) After one hour of isothermal hold at 20°C, the α‐phase forms. (c) After 18 h, the newly formed β′‐phase still coexists with the α‐phase. (d) The BuSP sample stored before X‐ray measurements at −18°C for 720 h (1 month) displays solely the β′‐phase. *Source*: Reused with permission from Pratama et al. (2022).

The DoC can also be determined from model fitting of the WAXS data (Arita‐Merino et al. 2020). In this approach, the area of the liquid fluid phase contribution and the area of all diffraction peaks are determined separately. An example of the fitting procedure is given in Figure 15 (Pratama et al. 2022), where all peaks were fitted with a Pearson VII function. The shape was fixed to a Gaussian (*m* = 100) for diffuse scattering and to a Lorentzian (*m* = 1) for fitting the diffraction peaks, which is the expected peak shape for the metastable phases in this example. The hexagonal and herringbone packing of hydrocarbon chains displays quasi‐long‐range order (Kuzmenko et al. 1998) as observed in the long tailing of diffraction peaks. This approach further allows estimating the fractions of each polymorphic form, simply by adding all diffraction areas associated with one phase, divided by the total scattering in the WAXS region (Arita‐Merino et al. 2020).

As mentioned before, the DoC values deduced from WAXS can be related to SFC values measured by NMR, which, within a 1% margin, reflect true solid percentages by weight (Gribnau, 1992). Thus, the group of Elke Scholten (Arita‐Merino et al. 2020) introduced a response factor method relating the measured diffraction areas associated with specific polymorphs to the respective mass fractions determined by NMR. Their method is an extension of the method introduced by Mazzanti et al. (2005) and Cisneros et al. (2006). We note that these correction factors differ from polymorph to polymorph and between fats. The response factors reported by Arita‐Merino et al. (2020), *F*_β′_, *F*_β_, and *F*_β′+β_, show that the combined response factor *F*_β′+β_ is not simply determined by the mean of the single response factors (*F*_β′_ and *F*_β_), but needs extra fitting. It is tempting to believe that this is due to the complex triglyceride composition of the AMF studied, leading to a β′ polymorphic form of different composition than the β′ form co‐existing with the β‐polymorphic form. Furthermore, the reported response factors for AMF and PO are not universal.

However, once determined, specific SFCs relating to individual polymorphs can be finally deduced. In any case, the method has its limits. As soon as the number of coexisting phases exceeds two, the determination of specific SFCs might become tedious, if not impossible, since two phases might have the same chain packing, but are actually composed of different TAGs with distinct crystal formation.

Another approach employing the Rietveld method was proposed by Calligaris et al. (2018). The authors quantified the β′ and β phases in binary blends of hydrogenated fats on the basis of already resolved β′ and β structures of the single TAGs PPS and SSS, respectively. While this approach allows determination of the response factors theoretically, it relies on available pure‐phase data and limited liquid contributions. As pointed out by Arita‐Merino et al. (2020), the Rietveld approach is limited to simple fats.

## 7 General Difficulties Encountered in the Analysis of Phase Transitions

Fat crystallization can take place in multiple steps depending on composition and process parameters. Fats containing a mixture of TAGs spanning a wide range of melting point temperatures often show superposition of fractional crystallization and polymorphic transitions (Himawan et al. 2006; Wesdorp et al. 2013). In a simple system with a pure component solid phase, a polymorphic transition from metastable (α) to stable crystal structure (β′, β) might occur. This can also be found for solid phases of miscible TAGs. The crystallization can either proceed as a solid–solid transformation or more easily as a melt‐mediated transition—generally known as a polymorphic transition (Sato et al. 2013). In contrast, a TAG mixture containing TAGs either assuming different lamellar stacking due to their molecular makeup and/or vastly different melting points might crystallize in multiple steps, that is, fractional crystallization. In this case, the crystallization events are more or less independent. In mixed systems, this can generate complicated crystallization pathways when these two phenomena are combined. This also commonly complicates the interpretation of SAXS and/or WAXS data.

In mixtures of truly immiscible components, the final diffractograms obtained from the respective pure components would be added up and scaled relative to their fraction in the mixture. However, in systems of structurally very similar TAG polymorphs, this results in superimposed diffraction peaks that might partially overlap. The analysis might be further hampered by the lower resolution of the lab‐scale instruments and diluted materials used. Here, the general inhomogeneity of a real fat system might result in diffraction peaks overlapping greatly, visible only by a shoulder contribution to a strong diffraction peak or in the apparent broadening of a diffraction peak stemming from two polymorphs with similar *d*‐spacings.

In the following, information obtained from SAXS and WAXS for binary mixtures and real fat systems and their dominant pure components are compared.

### 7.1 Information Obtained for Mixtures from SAXS

#### 7.1.1 Binary Mixtures

In a recent work, Cholakova et al. (2023) investigated the polymorphic crystallization of mixtures of monoacid TAGs. In detail, the 3^rd^ order SAXS reflections were resolved by fitting two Gaussian functions and then comparing them to the assumed lamellar stacking information for the pure components. This way, they were able to quantify the amount of each crystalline phase present. It is worth noting that all experiments were performed on a lab‐scale instrument and not at a synchrotron facility. It could be shown that mixtures of LLL and MMM do not co‐crystallize in the least stable polymorphic form, *α*, as observed for mixtures of other monoacid saturated triglycerides PPP and SSS (Himawan et al. 2007; MacNaughtan et al. 2006). It is plausible to be caused by the lower alkyl‐chain to glycerol ratio in LLL/MMM mixtures compared with PPP/SSS.

Immiscibility in structurally very similar molecules such as PPP and the molecular compound of POP and PPO (MC_POP/PPO_) poses a challenge for data interpretation. In a study on the phase behavior, the authors showed (note that they also used a lab‐scale instrument) two coexisting *β*‐phases via the 3^rd^ order reflection (Macridachis et al. 2022). Note, due to the inverse scattering angle to lattice spacing relationship in Bragg's law, superimposed diffraction peaks of higher orders are easier to resolve when two lamellar phases with similar lattices coexist. Utilizing analysis of the 3^rd^ order reflection is certainly not limited to binary mixtures. For example, Simone et al. (2024) used a second derivative approach in their studies on CBE and CBEs to disentangle polymorphic forms with nearly identical lattice spacings.

#### 7.1.2 Comparison of Pure Components and Real Fat Systems

The approach of calculating the EDP of a crystalline phase can also be applied to mixtures. TAG mixtures and complex fats have been analyzed regarding their polymorphic form and their lamellar spacing. In a mixture of SSS and OOO, mixed crystal formation in the α phase was reported (Mykhaylyk and Martin 2009). EDPs were also used to correlate the TAG composition of CBEs to the constructed EDPs and polymorphic crystallization tendencies in a study by Simone et al. (2024). In other studies, SAXS data were utilized to capture the polymorphic crystallization of common fats, including fully hydrogenated rapeseed oil (FHRO) (Seilert et al. 2024b), PO and AMF under shear (Mazzanti et al. 2005, 2009), and CB and CBE of different compositions (MacMillan et al. 2002; Simone et al. 2024; Yoshikawa et al. 2022).

Using the data from van Mechelen et al. (2006a) to generate EDPs, a comparison of POS and cocoa butter in the most stable polymorphic form can be made. The long spacings are 62.96 and 63.27 Å for POS and cocoa butter, respectively. Calculating the EDPs allows the determination of the bilayer and monolayer thickness. These are 38.48 and 23.79 Å for POS and 39.45 and 23.79 Å for cocoa butter. Further, for POS, a tilt angle of 33.2° and for cocoa butter of 32.1° were determined (both from β_2_, form V).

Another example is the comparison of cow and buffalo milk fat, where EDPs indicated a difference due to compositional differences (Pratama et al. 2021). BuSP makes up 3.93% and 4.57%, respectively, among a multitude of other TAGs, including mono‐unsaturated TAGs like POP, PSO, and SOS, and poly‐unsaturated TAGs like POO. For the β′‐3L polymorph of milk fat, the authors report a long spacing of 67.4 Å constituted of a bilayer thickness of 42.3 Å and a monolayer thickness of 25.1 Å. This is in good agreement with the values reported for SOS in the β′‐3L polymorph; see Table 5.

Differences in pure component and pseudo‐pure industrial‐grade material, SSS and FHRO, were recently communicated, exploring the crystalline state over various scales, USAXS, SAXS, and WAXS (Penagos et al. 2024a, 2024b). While the lamellar stacking (long spacing) was found to be comparable, 44.7 Å versus 44.8 Å, respectively, the crystalline nano platelet size (CNP), that is, single crystal size, was found to differ drastically. The CNP thickness derived from the Scherrer equation (Equation 4) gave 92.8 and 39.02 nm for SSS and FHRO, respectively. The authors assigned these drastic differences in this length scale to the impurities in the pseudo‐pure system FHRO, that is, the presence of other fatty acids besides stearic acid (∼ 90 %). Contrary to the more complex mixtures of cocoa butter and milk fat, this shows that a relatively pure blend of known composition shows comparable SAXS information to pure components. Note that the crystallization kinetics are not accounted for in this comparison, but it has been reported before that impurities in a system cause a delay in crystallization and polymorphic transition (Ray et al. 2013).

#### 7.1.3 Phase Transitions in Real Fat Systems

Phase transition of simple triglyceride systems, either pure or forming mixed crystals, can be followed by an increase in crystallinity or the evolution of the lamellar stacking, that is, *d*‐spacing. In fat systems of complex triglyceride composition, the phase change is less easy to determine. Some examples were reported by Tzompa‐Sosa et al. (2017) and Cisneros et al. (2006), investigating milk fat fractions, and Mazzanti et al. (2009) and Mazzanti et al. (2005) investigating milk fat and palm oil under shear, respectively. The latter group also reported about multistep turnovers during the crystallization process.

**FIGURE 16:**
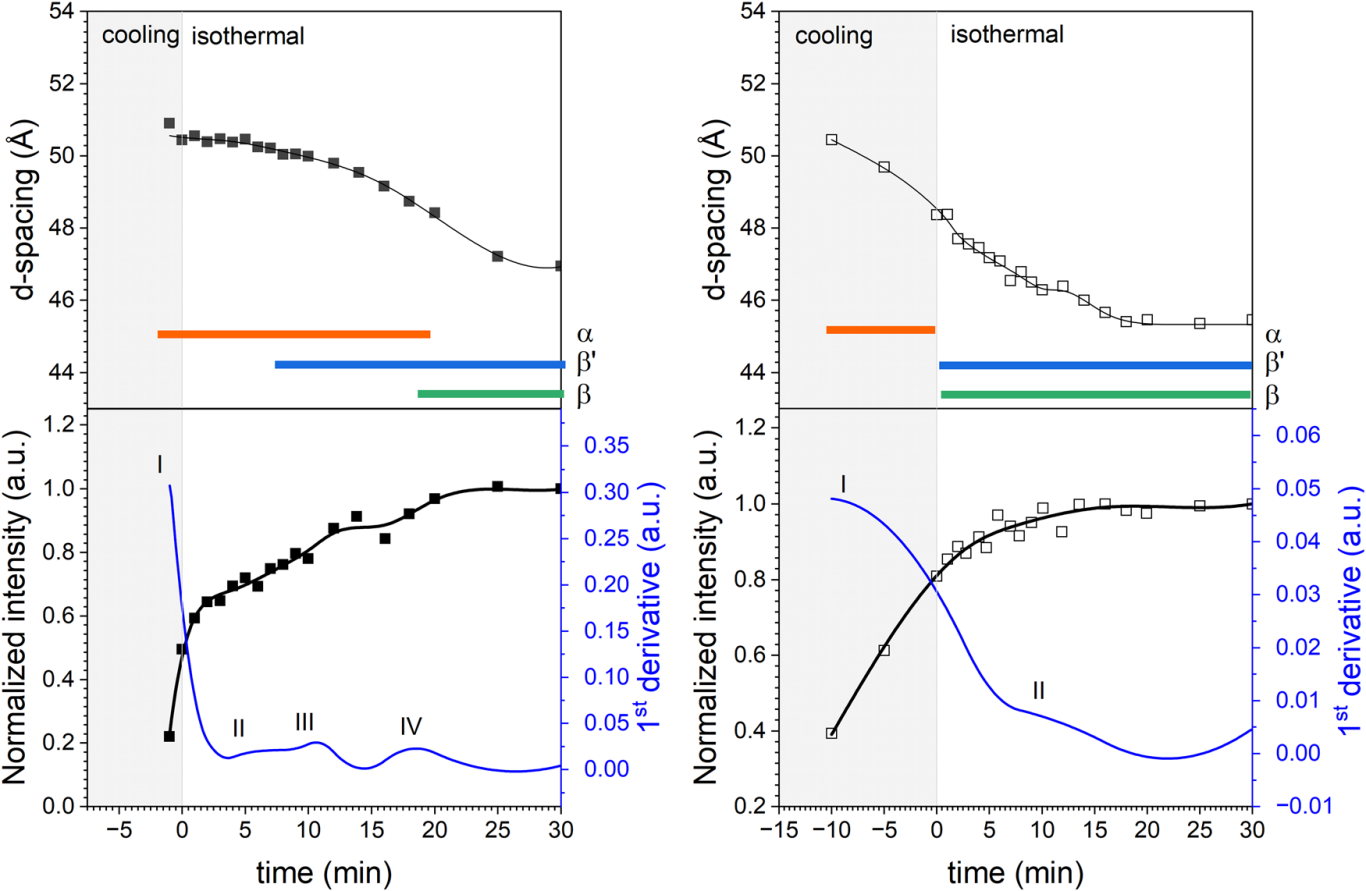
*d*‐spacing, normalized intensities in SAXD region and turnover curves (1st derivative), when crystallized at 5°C/min (left) and 1°C/min (right). Polymorphs as identified from the WAXD region in coloured bars (α orange, β′ blue, β green).

For example, a mixture of fractionated hydrogenated rapeseed oil and palm oil (FHRO/PO: 20/80% w/w), which was crystallized at a cooling rate of 5 and 1°C/min to 25°C and held isothermally for 30 min showed a slow restructuring process (slow decrease in *d*‐spacing) with clear identification of different polymorphic forms (Seilert et al. 2024a). When crystallized at a higher cooling rate, 5°C/min, an α phase was formed at first. This phase then co‐existed with a β phase that emerged. Only about 10 min later, a β′ phase was also identified. Yet, when crystallized at a low cooling rate (1°C/min) an α phase was detected and, as soon as the isothermal period started, β′ and β coexisted. Figure 16 shows the *d*‐spacings and the evolution of the crystallinity (normalized integrated peak intensity of the first‐order reflection). The first derivative of a spline interpolation allows the determination of different turnover events. The turnover curves align with phase changes/transitions determined from the WAXS region. The time scales do not necessarily align, as changes in the SAXS region might trail behind. This was recently observed for FHRO and its pure triglyceride SSS, where the transition from 2L(α) to 2L(β) took up to 8 h (Penagos et al. 2024b). Note, determining the first derivative of either the lattice spacing or intensity trends for analyzing multistep transitions has been proposed earlier by Rappolt and Rapp (1996).

The analysis is not limited to the SAXS region. A similar exercise can be performed on the integrated intensities of the WAXS region. This model‐free method proves particularly useful if material is analyzed that shows short spacings of presumably two different polymorphs, but the lamellar spacings show only marginal differences.

### 7.2 Information Obtained for Mixtures from WAXS

Besides the information derived from the SAXS region, assigning a polymorphic form from the WAXS region poses a challenge in mixtures. When comparing long spacing and crystalline nanoplatelet thickness, pure components and mixtures are difficult to compare in a detailed manner. Identifying the polymorphic phase information bases on generally‐agreed short spacings (AOCS Official method Cj 2–95(AOCS, 2003): α polymorph identified as a single peak at *d* = 4.15 Å, a β′ polymorph identified as two main peaks at *d* = 3.8 Å and *d* = 4.2 Å, and a β polymorph identified by a strong peak at around *d* = 4.6 Å and several other peaks). We note that the complexity in diffraction peaks for pure components, as documented in Table 7, is not considered in the above‐mentioned method.

In binary mixtures, the differences in short spacings with the mixing ratio are only minimal, as observed in binary mixtures of medium‐chain saturated TAGs (Danthine 2024). This also holds true for relatively pure real fat systems and their pure component counterparts, for example, FHRO and SSS. Cocoa butter and its equivalents made of shea butter, stearin, and palm mid fraction have been investigated in comparison to their constituting TAGs. POS dictates the WAXS fingerprint of cocoa butter as it is the dominating TAG (Ghazani and Marangoni 2019). Similar observations were communicated for shear stearin, whose polymorphic crystallization is dictated by SOS (Danthine et al. 2015) and CBEs, for which the phase behavior is directed by the most dominating TAG (Simone et al. 2024). Table 9 gives an overview of reported WAXS short spacings for pure components and related fat systems. Again, SSS and FHRO can be compared quite well, as FHRO consists mainly of stearic acid (∼90 %). With greater TAG diversity in real fat systems, it becomes more difficult to compare the WAXS deduced “fingerprints”.

**TABLE 9:** Summary of information derived from WAXS for pure components and related real fat systems.

		Area per chain (Å^2^)	Phase	Short spacings as reported (Å)	References
Pure component in dilution	SSS	18.76	β‐2L	4.6, 3.8, 3.7	Penagos et al. (2024a)
Fat system	FHRO	18.77	β‐2L	4.6, 3.8, 3.7	Penagos et al. (2024a)
Pure component	POS	19.12	β_1_‐3L	4.60, 4.04, 3.93, 3.86, 3.70	Ghazani and Marangoni (2019)
Fat system	Cocoa butter	19.31	β_1_‐3L (VI)	4.59, 4.04, 3.93, 3.86, 3.71, 3.56	Ghazani and Marangoni (2019)
Pure component	BuSP	18.9	β′‐3L	4.33, 4.14, 3.80	Pratama et al. (2022)
Pure component	PPO	19.4	β′_1_‐3L	4.2, 3.9	Macridachis et al. (2022)
Pure component	POO	20.7	β′‐3L	4.3, 4.1	Bayés‐García et al. (2016)
Fat system	Milk fat	18.85	β′‐2L and β′‐3L	4.15, 3.81	Pratama et al. (2021)
Fat system	Milk fat	20.21	β′‐2L	4.32, 3.89	Lopez et al. (2001)
Fat system	Milk fat	19.37	β′‐3L	4.19, 3.9	Lopez et al. (2001)
Pure component	SOS	n.d.	β′‐3L	4.30, 4.15, 4.02, 3.95, 3.83, 3.70	Sato et al. (1989)
Fat system	Shea Stearin in sunflower oil	n.d.	β′‐3L	4.36, 4.30, 4.17, 3.88, 3.80	Danthine et al. (2015)

*Note*: Short spacings and phases as reported in the respective reference and area per chain determined as described in this manuscript.

## 8 Conclusion and Future Research Challenges

In this review, we explored the structural similarities in the nanostructural packing and stacking information deduced from X‐ray scattering of triglyceride crystals. Literature data on various TAG groups, for example, monoacid and mixed‐acid saturated, and mono‐unsaturated, have been analyzed. Trends in packing geometries at the smallest scale, that is, 3D‐subcell packing, and larger scale, lamellar stacking, and chain tilt angle evaluation have been discussed in great depth.

Section 3 presents a literature review on molecular structures in triglyceride crystals, which explains the triglyceride polymorphism, the role of the glycerol moiety, and unit cell information. The chain orientation is summarized in detail in three main arrangements, namely the α, β′, and β polymorphs. Interestingly, the orientation of the glycerol moiety, that is, tuning fork or chair, is dependent on the polymorphic form for saturated TAGs (chair in β′ and tuning fork in β). In unsaturated TAGs, the unsaturated fatty acid chain dictates the glycerol configuration. Differences in systematics are also revealed in the available unit cell information.

In Section 4, the nanostructural stacking information deduced from SAXS is reviewed. The calculation of electron density profiles and the chain tilt angle determination is illustrated for homologous series of saturated and mono‐unsaturated TAGs. Further, information obtained from the peak width, that is, crystallite size and strain, has been summarized. In Section 5, the nanostructural packing information deduced from WAXS is summarized and reviewed, including a discussion on the 3D‐subcell information. A comprehensive list of short spacings for various TAGs is provided.

Section 6 presents available methods to determine the solid fat content of real fat systems via model‐free WAXS analysis and a WAXS model fitting approach. Section 7 concludes with the translation from pure component data into real fat systems and the complexity encountered when dealing with systems undergoing phase transition in situ. The challenging task of relating pure component properties to real fat systems is carried out by discussing cocoa butter and milk fat, among others.

This review summarizes quite extensively the advances of X‐ray scattering methods applied to the field of TAG's research, and still, various open questions remain to be addressed. First, it is highly desirable to be able to determine the fractions of coexisting polymorphs. This can either be achieved by a rigorous crystallographic analysis of coexisting phases, for example, by extending the commonly applied Rietveld method for pure crystalline substances (Rietveld 2010) to mixtures thereof. Alternatively, the small‐angle X‐ray diffraction regime can be exploited to estimate volume fractions of coexisting polymorphs. As demonstrated in a recent study (Ladd Parada et al. 2018), global analysis techniques enable interpolation of diffraction data for each coexisting polymorph to the scattering angle of zero (*q* = 0). Here, one can make use of the fact that different form factor contributions to the intensity *I(0)* scale with their respective volumes. Second, progress has been recently made in characterizing the fluid phase of TAGs, understanding more deeply the formation of the back‐to‐back clustering of triglycerides (Sadeghpour et al. 2018; Tascini et al. 2018; Golodnizky et al. 2022; Mazzanti et al. 2024). However, it would be further useful to know the fraction of clustered TAGs within the fluid phase to classify TAG‐blends by the amount and specific structure of the formed clusters, which are hypothesized to influence the nucleation and growth mechanism of food fat crystals. Third, the type of underlying lamellar disorder of the different polymorphic crystals is scarcely studied. As recently pointed out by Povey and Hefft (2023), the meta‐stable polymorphs of the α‐ and β′ phase are mechanically behaving as soft solids, which is also reflected in the quasi‐long‐range order of their lamellar stacking. This is contrasted by the given long‐range order in the stable β′ and β crystals of pure TAG systems, where only thermal disorder is apparent. Thus, which role mechanical differences of the polymorphs may play in the formulation of real fat systems is still to be investigated in depth.

In conclusion, this work provides an extensive overview of crystallographic information obtained from literature spanning several decades of research. Further, data analysis methods are revisited and discussed that allow following the phase transition and the estimation of the solid fat fraction in complex fat systems. Finally, this contribution highlights practical methods available for successful evaluation of the polymorphic state and phase transition by means of X‐ray scattering to aid research efforts at the interface of academia and industry.

## Author Contributions

**Julia Seilert**: conceptualization, investigation, writing–review and editing, writing–original draft, data curation, resources, formal analysis, software. **Megan Holdstock**: writing–review and editing, data curation, investigation, formal analysis, methodology. **Yoga Pratama**: investigation, data curation. **Amin Sadeghpour**: data curation, investigation, methodology. **Eckhard Flöter**: conceptualization, writing–review and editing. **Michael Rappolt**: conceptualization, investigation, writing–original draft, writing–review and editing, supervision, data curation, software, formal analysis, methodology.

## Conflicts of Interest

The authors declare no conflicts of interest.

## Supporting information




**Supplementary Materials**: crf370271‐sup‐0001‐SupMat.docx
