# Supplementary material for: Polymorphic Structures and Transitions of Triglycerides and Complex Fats: A Guide to X‐Ray Scattering Approaches
Source: Compr Rev Food Sci Food Saf. 2025 Oct 5;24(6):e70271. doi: 10.1111/1541-4337.70271 (PMC12497477; doi:10.1111/1541-4337.70271)
Supplement: Supplementary file 1 — Supplementary Materials: crf370271‐sup‐0001‐SupMat.docx [file CRF3-24-e70271-s001.docx]

**Supporting Information**

**Polymorphic Structures and Transitions of Triglycerides and Complex Fats: a Guide to X-ray Scattering Approaches**

Julia Seilert^a^, Megan Holdstock^b^, Yoga Pratama^b, c^, Amin Sadeghpour^b^, Eckhard Flöter^a^, and Michael Rappolt^b*^

^a^ Department of Food Process Engineering, Technische Universität Berlin, Straße des 17. Juni 135, Berlin 10623, Germany

^b^ School of Food Science and Nutrition, University of Leeds, LS2 9JT, U.K.

^c^ Current address: Department of Food Technology, Faculty of Animal and Agricultural Sciences, Universitas Diponegoro, Semarang 50275, Indonesia

*Corresponding author: e-mail m.rappolt@leeds.ac.uk

**Keywords**: Triglycerides, Polymorphs, Phase Transition, Solid Fat Content, X-ray Scattering

**S1. Electron Density Profiles for Selected Triglycerides in Different Polymorphic Forms.**


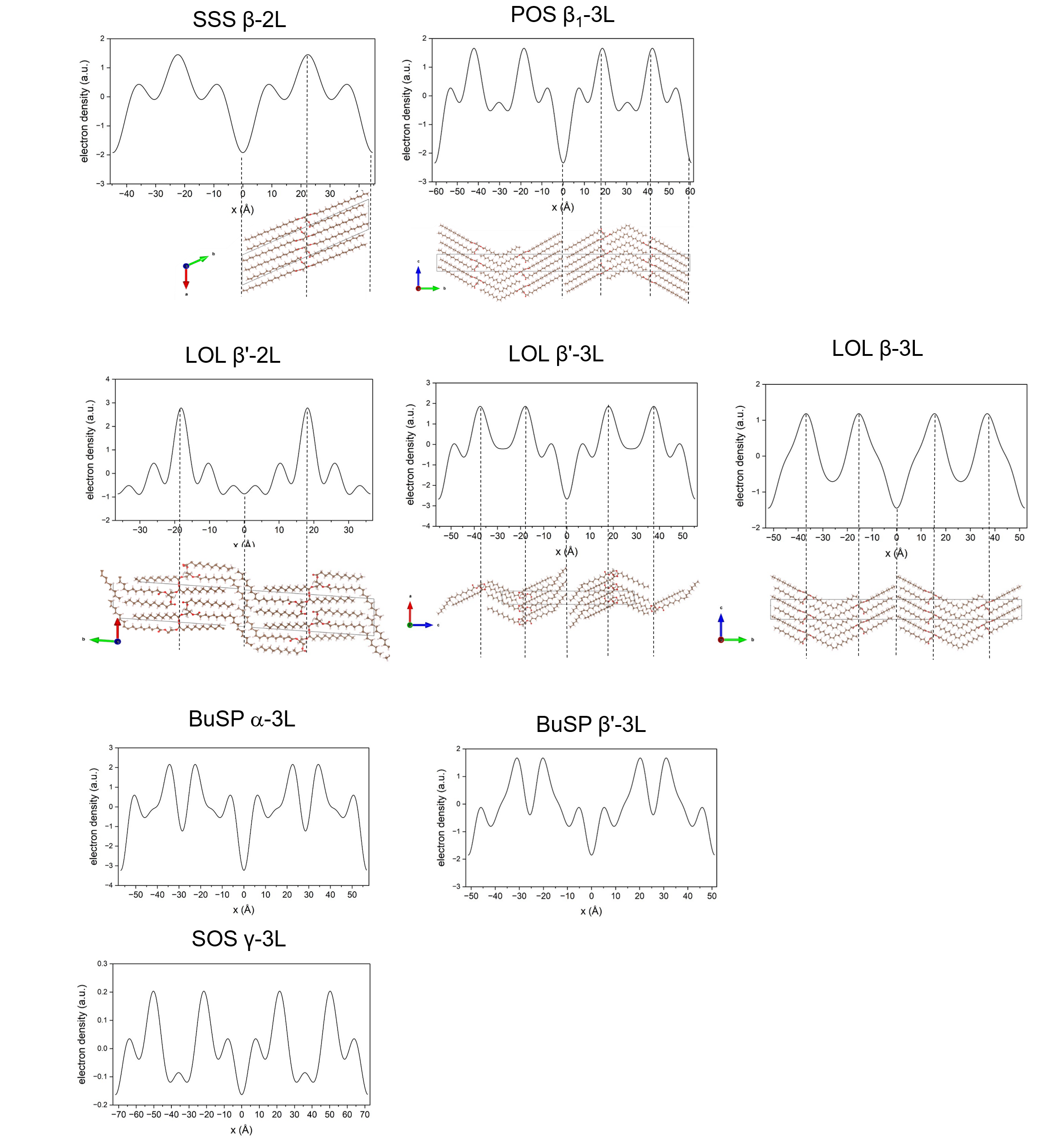


**Figure S1.** EDPs for SSS in β-2L (van Langevelde et al. 2001), POS in β1-3L (van Mechelen et al. 2006), SOS γ-3L , LOL in β’-2L, β’-3L and β-3L (van Mechelen et al. 2008), and BuSP in α-3L and β’-3L (Pratama et al. 2022) and visualization of the triglyceride unit cell if crystal structure where available generated using VESTA (Momma and Izumi 2011).

**
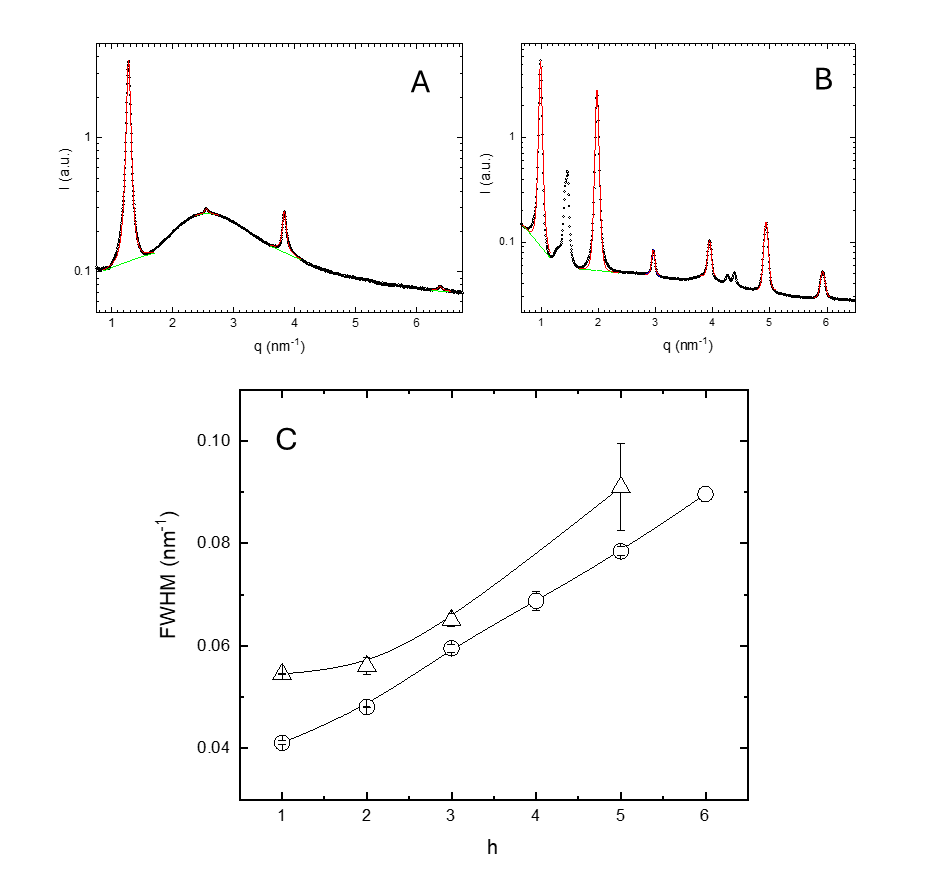
S2. Full Width Half Maximum Analysis of the α and β Phase of Cocoa Butter**

**Figure S2.** The FWHM analysis of cocoa butter demonstrates that only quasi long-range order is provided in the stacking of lamellae in the α and β phase. (A) Diffraction pattern of the α polymorph is shown (data taken from Ladd Parada et al. 2018), and the 1^st^, 2^nd^, 3^rd^ and 5^th^ order peak were fitted with Pearson VII functions (red line) and the background was fitted with a 2^nd^ degree polynomial (green line). (B) Accordingly, the diffraction pattern of the β polymorph is presented (data taken from Simone et al. 2024), and the 1^st^ to 6^th^ order peak were fitted with Pearson VII functions (red line) and the background was modelled with a 2^nd^ degree polynomial (green line). Note, the 3L-β phase does coexist with traces of two distinct 2L-β phases. (C) FWHMs as a function of the Miller index h are displayed (Δ: α phase; o: β phase), clearly demonstrating stacking disorder of second kind, since the FWHM are increasing with the Miller index (Pabst 2006). Note, thermal disorder would display constant FWHM for all diffraction orders.

**S3. Refinement of the Glycerol Backbone Extension in 2L- and 3L-Phases**

We have determined the exact chain tilt of the chains for nine different data sets available in VESTA (Momma and Izumi 2011), namely three 2L-β phases from MMM, PPP and SSS, and three 3L- β_2_ and three 3L- β_1_ phases from POP, POS and SOS, respectively. For sake of clarity, the methyl end plane shall be defined with the **a** and **b** unit cell vectors (note, this might be different in the published data sets). The vector normal to the **a**, **b** plane, we shall call **n**. Now VESTA permits to determine the projected tilt angles, θ_a_ and θ_b_ with respect to **n** in the **a**, **n** and **b**, **n** plane, respectively. Thus, we can express the coordinates of the chain-axis vector **v** = (tan θ_a_, tan θ_b_, 1) given in the coordinate system **a**, **b** and **n**. It can be shown by the vector product of **v** and **n** that the overall tilt angle, θ, in the coordinate system of **a**, **b** and **n** is given by:

$\tan\left( \theta\right)= \sqrt{{tan}^{2}{(\theta}_{a})+ {tan}^{2}(\theta_{b})}$ **Eq. S1**

We note, that for **a** and **b** being perpendicular to each other Eq. S1 delivers directly the overall chain tilt angle. For **a** and **b** including the angle γ ≠ 90° a transformation to Cartesian coordinates is necessary, with a* = a cos (90°-γ) and b* = b – sin(90°-γ).

In summary, we obtained overall tilt angles of 30.1°, 29.7°, and 29.9° for MMM, PPP, ad SSS, respectively. For the three 3L- β_2_ and three 3L- β_1_ phases from POP, POS and SOS we determined tilt angles of 33.1°, 33.2°, 33.8° and 33.2°, 33.2°, 33.1°. From these fiducial tilt angle values deduced from crystallographic data, we then refined the glycerol backbone extension in the 2L-stacking to take a value of **2.54 Å** (cp. **Eq. 2**) and in the 3L-stacking to take a value of **1.54 Å** (cp. **Eq. 3**).

**References**

Ladd Parada, M., Sadeghpour, A., Vieira, J., Povey, M., and M. Rappolt. 2018. “Global Small-Angle X-ray Scattering Data Analysis of Triacylglycerols in the α-Phase (Part II).” *The Journal of Physical Chemistry. B* 122, no. 45: 10330–10336. https://doi.org/10.1021/acs.jpcb.8b06708

Momma, K., and F. Izumi. 2011. “VESTA 3 for three-dimensional visualization of crystal, volumetric and morphology data.” *Journal of Applied Crystallography* 44, no. 6: 1272–1276. https://doi.org/10.1107/S0021889811038970

Pabst, G. 2006. “Global properties of biomimetic membranes: perspectives on molecular features.” *Biophysical Reviews and Letters* 1, no. 11: 57–84.

Pratama, Y., Burholt, S., Baker, D. L., Sadeghpour, A., Simone, E., and M. Rappolt. 2022. “Polymorphism of a Highly Asymmetrical Triacylglycerol in Milk Fat: 1-Butyryl 2-Stearoyl 3-Palmitoyl-glycerol.” *Crystal Growth & Design* 22, no. 10: 6120–6130. https://doi.org/10.1021/acs.cgd.2c00713

Simone, E., Rappolt, M., Ewens, H., Rutherford, T., Marty Terrade, S., Giuffrida, F., and C. Marmet. 2024. “A synchrotron X-ray scattering study of the crystallization behavior of mixtures of confectionary triacylglycerides: Effect of chemical composition and shear on polymorphism and kinetics.” *Food Research International* 177, art. number 113864. https://doi.org/10.1016/j.foodres.2023.113864

van Mechelen, J. B., Goubitz, K., Pop, M., Peschar, R., and H. Schenk. 2008. “Structures of mono-unsaturated triacylglycerols. V. The β′ 1 -2, β′-3 and β 2 -3 polymorphs of 1,3-dilauroyl-2-oleoylglycerol (LaOLa) from synchrotron and laboratory powder diffraction data.” *Acta Crystallographica Section B Structural Science* 64, no. 6: 771–779. https://doi.org/10.1107/S0108768108031601

van Mechelen, J. B., Peschar, R., and H. Schenk. 2006. “Structures of mono-unsaturated triacylglycerols. I. The β 1 polymorph.” *Acta Crystallographica Section B Structural Science* 62, no. 6: 1121–1130. https://doi.org/10.1107/S0108768106037074

van Langevelde, A., Peschar, R., and H. Schenk. 2001. “Structure of β-trimyristin and β-tristearin from high resolution X-ray powder diffraction data.” *Acta Crystallographica Section B Structural Science* B57: 372–377.
